# Viroporin activity is necessary for intercellular calcium signals that contribute to viral pathogenesis

**DOI:** 10.1126/sciadv.adq8115

**Published:** 2025-01-17

**Authors:** J. Thomas Gebert, Francesca J. Scribano, Kristen A. Engevik, Ethan M. Huleatt, Michael R. Eledge, Lauren E. Dorn, Asha A. Philip, Takahiro Kawagishi, Harry B. Greenberg, John T. Patton, Joseph M. Hyser

**Affiliations:** ^1^ Department of Molecular Virology and Microbiology, Baylor College of Medicine, Houston, TX 77030, USA.; ^2^ Alkek Center for Metagenomics & Microbiome Research, Baylor College of Medicine, Houston, TX 77030, USA.; ^3^ Department of Biology, Indiana University, Bloomington, IN 47405, USA.; ^4^ Departments of Medicine and Microbiology and Immunology, Stanford University School of Medicine, Stanford, CA 94305, USA.

## Abstract

Viruses engage in a variety of processes to subvert host defenses and create an environment amenable to replication. Here, using rotavirus as a prototype, we show that calcium conductance out of the endoplasmic reticulum by the virus encoded ion channel, *NSP4*, induces intercellular calcium waves that extend beyond the infected cell and contribute to pathogenesis. Viruses that lack the ability to induce this signaling show diminished viral shedding and attenuated disease in a mouse model of rotavirus diarrhea. This implicates nonstructural protein 4 (NSP4) as a virulence factor and provides mechanistic insight into its mode of action. Critically, this signaling induces a transcriptional signature characteristic of interferon-independent innate immune activation, which is not observed in response to a mutant NSP4 that does not conduct calcium. This implicates calcium dysregulation as a means of pathogen recognition, a theme broadly applicable to calcium-altering pathogens beyond rotavirus.

## Teaser

The NSP4 viroporin initiates an innate immune response and intercellular calcium waves that contribute to pathogenesis.

## INTRODUCTION

Acute gastroenteritis (AGE) remains a leading cause of mortality and morbidity among young children worldwide (*1*). Viruses are the most common enteric pathogens, with rotavirus (RV) alone accounting for one quarter of all cases of severe pediatric AGE (*2*, *3*). Viral AGE typically presents with watery diarrhea, vomiting, fever, abdominal pain, and malaise (*4*). While most cases of viral AGE are self-limiting, AGE can progress to cause life-threatening dehydration and volume depletion. For these cases, oral rehydration therapy (ORT) is the standard of care (*5*). ORT has tremendously reduced the mortality of AGE and all-cause childhood mortality as a result. However, still today nearly 500,000 children die from AGE annually (*2*). While effective to combat dehydration, ORT offers symptomatic treatment without addressing the underlying infection. Hence, ORT does not prevent viral spread or outbreaks.

In addition to ORT, the development of live-attenuated RV vaccines has helped to reduce the burden of AGE worldwide. RV has long been the most common cause of AGE in children. Both clinical trials and real-world efficacy studies suggest that in the United States, RV vaccines are around 85% effective in the prevention of RV-associated hospitalizations (*6*, *7*). Unfortunately, this degree of efficacy has not been reproduced in areas of the world with higher rates of all-cause childhood mortality. While RV vaccine efficacy is about 86% in countries with low childhood mortality, it falls to only 54 to 63% in countries with moderate and high rates of childhood mortality (*8*). Improved vaccines will be integral to combat RV globally, and achieving this goal will require a better understanding of the molecular mechanisms of RV pathogenicity. Thus far, all licensed RV vaccines have been based on live-attenuated strains (*9*). While multiple groups are working to develop subunit, mRNA, and inactivated RV vaccines, these efforts have yet to show adequate efficacy in clinical trials. A recently developed subunit vaccine based on the RV spike protein, VP8, failed to meet efficacy criteria in initial stages of clinical trials, and further development was halted (*10*). While other candidates have shown promise in preclinical studies, live-attenuated vaccines (LAVs) remain the most effective tool for conferring protection against RV.

Despite the widespread use of LAVs, we have yet to fully comprehend the molecular mechanisms that allow for RV attenuation. Hence, the development of LAVs for RV has relied on empirical assessment of cell culture–adapted or naturally circulating attenuated strains (*11*, *12*). The advent of the reverse genetics system for RV offers a promising alternative for vaccine development (*13*). Currently, simian, some human, and murine-like recombinant RV strains show efficient rescue with the existing reverse genetics strategies, and there have been recent advances toward a higher efficiency human RV reverse genetics system (*14*, *15*). Hence, there is potential to intelligently engineer an improved live-attenuated strain. This could allow for attenuation of virulence without major changes to viral antigenicity or fitness, ultimately improving immunogenicity and protection. However, without a comprehensive understanding of RV virulence factors, such a rational design is unlikely to generate the desired attenuated traits.

The virulence of RV disease is multigenic, having been associated with 5 of the 11 RV genes. These include the genes for the capping enzyme and interferon antagonist, VP3, the outer capsid proteins VP4 and VP7, the interferon-antagonizing protein NSP1 (nonstructural protein 1), and the multifunctional NSP4 (*16*, *17*). Broadly, RV virulence is proportional to the severity of diarrhea it causes. RV-induced diarrhea is multifactorial, driven both by malabsorption and hypersecretion (*18*). Our understanding of RV disease evolved upon the discovery of the enterotoxin function of NSP4 (*19*). In a 1996 study, investigators found that intraperitoneal injection of purified NSP4 was sufficient to induce diarrhea in mouse pups (*19*). This study established NSP4 as the first known enterotoxin of viral origin. However, studying the potential enterotoxin activity of NSP4 during a bona fide RV infection has proven more challenging. While the enterotoxin NSP4 is likely a key mediator of RV disease, we have yet to determine its relative contribution to the overall diarrhea phenotype. NSP4 has since been found to also function intracellularly as a viroporin, forming a calcium (Ca^2+^)–conductive ion channel that releases Ca^2+^ from the endoplasmic reticulum (ER) store (*20*, *21*). NSP4 variants associated with attenuated diarrhea induction in vivo show a diminished capacity to increase cytosolic Ca^2+^ when expressed in Sf9 cells, suggesting a role for NSP4 Ca^2+^ mobilization in virulence (*22*).

We recently found that RV-infected cells release adenosine diphosphate (ADP) in pulses throughout infection, constituting a previously unidentified contributor to RV virulence (*23*). By activating the P2Y1 receptor, extracellular ADP drives an increase in cytosolic Ca^2+^ in neighboring, uninfected cells, producing a signal known as an intercellular Ca^2+^ wave (ICW). Blocking the P2Y1 receptor, as well as the resulting ICWs, reduces disease severity in neonatal mouse pups infected with a heterologous (simian) RV strain. Furthermore, preventing ICWs reduces viral spread in cell culture, suggesting that they contribute to the formation of a viral replication niche or microenvironment (*24*). Knockdown of NSP4 reduces the frequency of ICWs from RV-infected cells, suggesting that it may be involved in ICW induction. However, knockdown of NSP4 affects the expression and distribution of multiple other viral proteins and impairs particle assembly; thus, the effect of NSP4 knockdown on ICWs may be indirect (*25*).

Given these findings, it is likely that ICWs contribute to the establishment of a viral replication niche and, ultimately, to RV virulence; however, we have yet to fully characterize the viral determinants of this signaling. In this study, we used existing human and porcine RV strains, as well as novel recombinants generated by the RV reverse genetics system, to examine the role of NSP4 in the induction of ICWs using in vitro, organoid, and in vivo model systems. Ultimately, we found that the ability of RV to generate ICWs segregates with NSP4, expression of NSP4 alone is sufficient to generate ICWs, and multiple aspects of RV disease severity correlate with the ability to generate ICWs. Overall, this work implicates NSP4 viroporin activity as the primary driver of RV-induced ICWs and a critical component of the pathogen-induced changes in host cell physiology.

## RESULTS

### Virulent porcine RV strain induces a higher frequency of ICWs than its attenuated counterpart

**Fig. 1.**
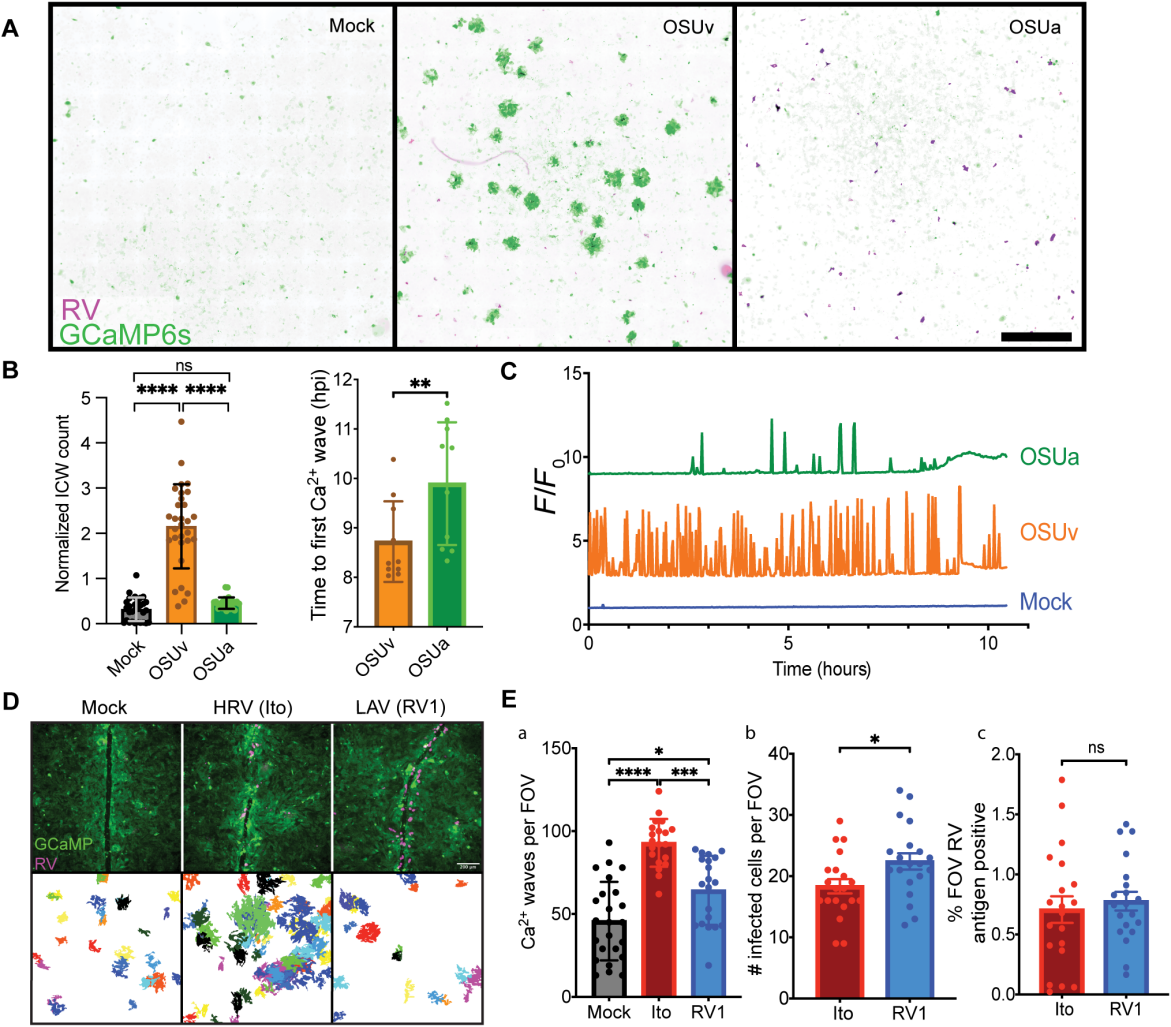
Frequency of ICWs is associated with virulence. (**A**) Maximum intensity projections of MA104-GCaMP6s monolayers imaged after infecting with mock inoculum, OSUv, or OSUa RV at an MOI of 0.01. ICWs are indicated by regions of high GCaMP6s intensity (green) surrounding RV-infected cells (magenta), seen predominantly in the OSUv-infected monolayer. Regions of each monolayer measuring 10 mm by 10 mm were imaged for 30 min beginning 18 hpi. (**B**) MA104-GCaMP6s monolayers were infected at an MOI of 0.005 and imaged from 8 to 24 hpi. ICWs were quantitated using an automated pipeline developed in FIJI. The relative quantity, normalized against the mean ICW count in mock-infected monolayers for a given replicate (left) and latency (right) of ICWs between mock, OSUv, and OSUa infection. Data are pooled from three biological replicates. (**C**) Representative intracellular Ca^2+^ traces from mock-, OSUv-, or OSUa-infected cells. (**D**) Representative HIO monolayers expressing GCaMP6s (green) and infected with mock inoculum**,** virulent human RV, or an attenuated RV strain. Monolayers were imaged from 8 to 24 hpi before fixing and staining for RV antigen (magenta). ICWs detected over the course of imaging were arbitrarily pseudocolored and projected into a single image for each multipoint. (**E**) The quantity of ICWs (a) and infected cells (b) and the percentage of monolayer area positive for RV antigen compared between mock, Ito, and RV1 infection (c). Data are combined from three biological replicates. Data in bar charts represent mean with error bars denoting SD. *P < 0.05, **P < 0.01, ***P < 0.001, and ****P < 0.0001. Outliers were removed using the ROUT method (*Q* = 1%) and normality determined by Shapiro-Wilk tests. (B) (left) and (E) (a) were analyzed using Kruskal-Wallis, followed by Dunn’s multiple comparison tests. (B) (right) and (E) (b and c) were analyzed using Mann-Whitney tests. FOV, field of view; ns, not significant.

On the basis of our previous studies that demonstrated that pharmacologic blockade of ADP-mediated ICWs lessened disease severity in RV-infected mice (*23*), we sought to determine whether attenuated RV strains elicit fewer ICWs during infection. We performed long-term Ca^2+^ imaging on monolayers infected with RV strains of varying virulence. For these studies, we first investigated the porcine RV strains OSUv and OSUa (*25*). OSUv was isolated from a sick neonatal piglet and was serial passaged in cell culture to generate the attenuated strain, OSUa (*22*, *26*–*28*). Upon imaging, we found that cells infected with OSUv induced more ICWs than OSUa-infected cells (Fig. 1, A to D). This was quantifiable by two distinct methods. First, we infected monolayers of MA104 cells expressing GCaMP6s (MA104-GCaMP6s) with the indicated RV strains at a multiplicity of infection (MOI) of 0.01. For each infection, we imaged a 10-mm by 10-mm stitched field of view once every 2 min for 30 min beginning at 18 hours postinfection (hpi). We then generated maximum intensity projections of each field of view, allowing for visualization of all ICWs detected in the 30-min period (Fig. 1A). In addition, to capture a longer duration of the infection, we infected MA104-GCaMP6s monolayers with the indicated RV strains at an MOI of 0.01 and imaged eight positions per monolayer every 1 min from 8 to 24 hpi. We developed an analysis pipeline in FIJI that allowed for automated quantitation of ICWs detected within each field of view during the 16 hours of imaging. Using this approach, we detected a fourfold increase in ICWs in monolayers infected with OSUv compared to those infected with either mock inoculum or OSUa (Fig. 1B). Furthermore, the ICWs from OSUv-infected cells began earlier after infection than those from OSUa-infected cells (Fig. 1B). The overall increase in Ca^2+^signaling associated with OSUv is further illustrated by Ca^2+^ traces from representative mock-, OSUv-, and OSUa-infected cells (Fig. 1C). Thus, the virulent strain, OSUv, and the closely related attenuated strain, OSUa, have distinct ICW phenotypes despite strong homology, offering an ideal pair of viruses to investigate the genetic determinants of RV-induced ICWs.

### Virulent human RV strain induces higher frequency of ICWs than live-attenuated strain

Given the association between virulence and ICWs observed with the porcine RV strains, we next aimed to determine whether this relationship would hold among human RV strains. Using a similar experimental approach, we infected monolayers of jejunal human intestinal organoids (jHIOs) engineered to express the cytosolic calcium indicator GCaMP6s (jHIO-GCaMP6s) with virulent or attenuated human RV strains. For the virulent strain, we selected Ito, as it was previously shown to infect HIOs and induce ICWs (*23*, *29*). For the attenuated strain, we selected RV1, which is one of the most widely used LAVs (ROTARIX) (*9*). Representative fields of view for mock-, Ito-, and RV1-infected jHIO-GCaMP6s monolayers show the expected detection of RV near the site of a monolayer scratch (Fig. 1D). The ICW phenotypes associated with each virus are illustrated by the projection of all ICWs detected in each field of view over the preceding 18 hours of imaging, which have been pseudocolored to distinguish individual ICW events (Fig. 1D). Quantification of ICWs detected across all fields of view confirmed that, consistent with observations using the porcine strains, the virulent human strain, Ito, induced significantly more ICWs during infection than the attenuated strain, RV1 (Fig. 1Ea). To determine whether this may have been due to increased infectivity of the virulent strain, we fixed monolayers after imaging and detected RV antigen by immunofluorescence. We quantitated the number of infected cells per field of view (Fig. 1Eb) and the percentage of each field of view that was antigen positive after flat-field correction, background subtraction, and Otsu thresholding (Fig. 1Ec) (*30*). The number of infected cells was slightly higher in monolayers infected with RV1 (18.4 versus 22.4 for Ito and RV1, respectively; Fig. 1Eb). We detected no difference in the percentage of the monolayer area that was antigen positive (Fig. 1Ec). Together, the greater number of ICWs in monolayers infected with the virulent human strain, Ito, was not attributable to increased infectivity.

### ICW phenotype segregates with gene 10, encoding NSP4

**Fig. 2.**
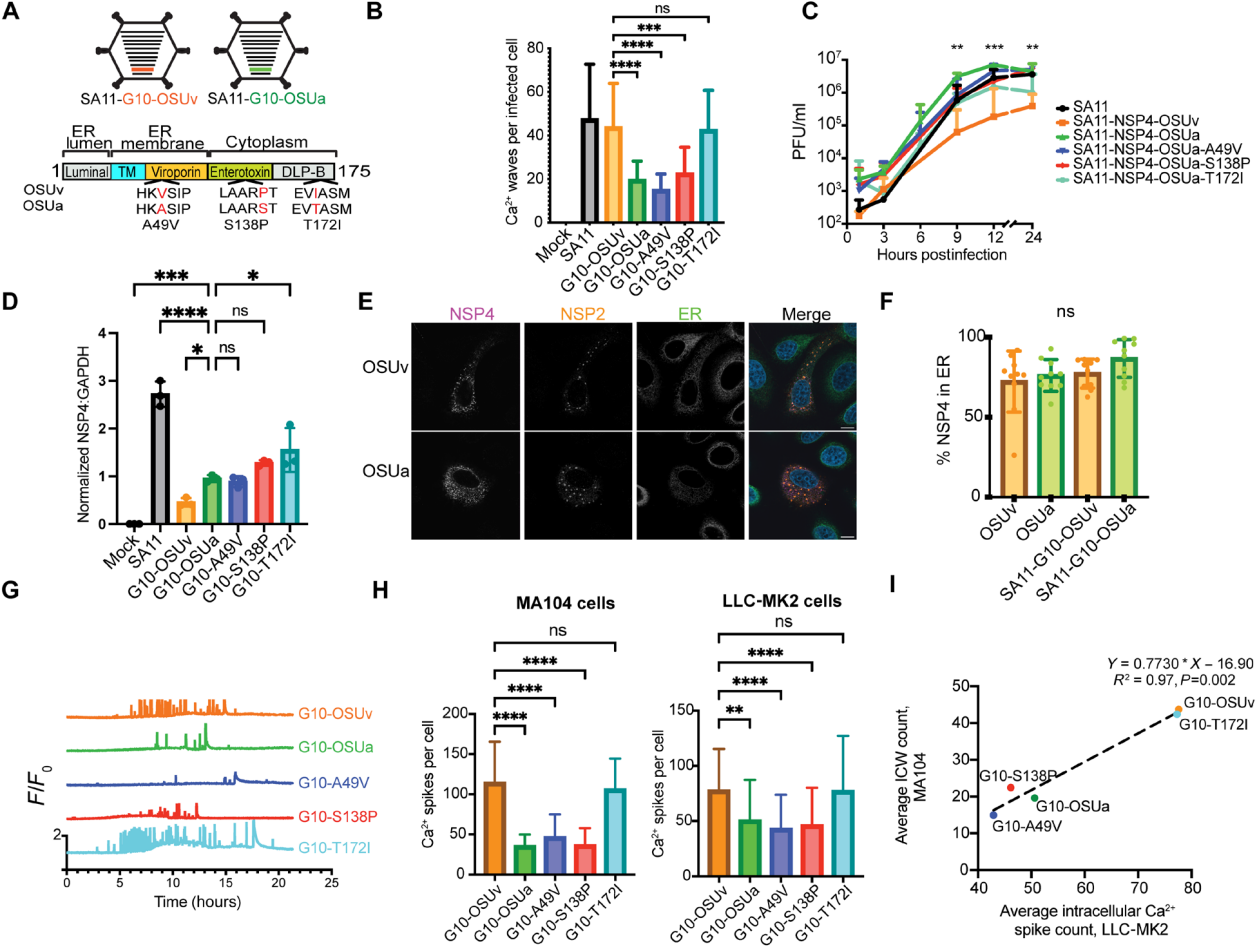
The difference in ICW phenotypes is attributable to NSP4. (**A**) Recombinant SA11 strains with NSP4 from OSUv or OSUa and map of NSP4 highlighting the main domains [luminal, transmembrane (TM), viroporin, enterotoxin, and DLP-B], their location in the ER lumen, membrane or cytoplasm, and the three amino acid differences between OSUv and OSUa NSP4s. (**B**) ICW quantification in MA104-GCaMP6s monolayers infected with mock inoculum, wild-type SA11, or SA11 encoding G10 (NSP4) from OSUv, OSUa, or the indicated single amino acid revertants from OSUa to OSUv. Monolayers were imaged from 8 to 24 hpi and ICWs quantitated across three biological replicates. ICW counts were compared by Kruskal-Wallis test, followed by Dunn’s multiple comparison. (**C**) Growth curves of virus replication kinetics. Asterisks indicate Mann-Whitney *U* tests comparing SA11-G10-OSUv and SA11-G10-OSUa at the indicated time points. (**D**) Normalized NSP4:glyceraldehyde-3-phosphate dehydrogenase (GAPDH) ratio at 9 hpi in MA104 monolayer lysate following infection at an MOI of 5 with the indicated virus. Relative quantities compared by one-way analysis of variance (ANOVA), followed by Holm-Šídák tests. (**E**) Representative images of SA11-G10-OSUv and SA11-G10-OSUa infected monolayers expressing ER-localized GFP (green) with NSP4 (magenta) and NSP2 (orange) detected by immunofluorescence at 12 hpi. Scale bars, 10 μm. (**F**) Mander’s coefficient to estimate NSP4 colocalization with Sec61β as a surrogate for ER localization. Data are combined from 15 cells across three biological replicates. Groups compared by Kruskal-Wallis test (*P* > 0.05). (**G**) Representative intracellular Ca^2+^ traces from cells infected with the indicated strain of recombinant SA11. (**H**) Intracellular Ca^2+^ spike counts from MA104-GCaMP6s (left) or LLC-MK2-GCaMP6s (right) cells (ICW-deficient) infected with the indicated strain of recombinant SA11. Data are combined from three biological replicates. ICWs were compared by Kruskal-Wallis test, followed by Dunn’s multiple comparison tests. (**I**) Linear regression estimating the association between intracellular Ca^2+^ spike counts in LLC-MK2–GCaMP6s cells and ICW counts in MA104-GCaMP6s cells. *P < 0.05, **P < 0.01, ***P < 0.001, and ****P < 0.0001.

We next aimed to determine which viral protein(s) may drive the observed difference in ICW phenotype. For these experiments, we focused on OSUv and OSUa, as high sequence homology facilitates phenotype-genotype mapping (*22*, *31*). In previous studies, we found that abrogation of NSP4 expression using a small-hairpin RNA reduced the number of ICWs that occur during infection (*23*). On this basis, we hypothesized that differences in NSP4 account for the observed differences in the ICW phenotype between OSUv and OSUa. To isolate the effects of the changes in NSP4 between OSUv and OSUa, we generated two novel RV strains using the reverse genetics system, both in an simian agent 11 (SA11) simian RV background. In the first strain, we replaced SA11 gene 10, which encodes NSP4, with that from OSUv (SA11-G10-OSUv). In the second, we replaced gene 10 with that from OSUa (SA11-G10-OSUa) (Fig. 2A). Only three amino acids are different in the NSP4 protein from OSUv and OSUa and, on the basis of our previously proposed map of NSP4 domains, these differences map to the viroporin domain (A49V), the enterotoxin domain (P138S), and the double-layered particle binding (DLP-B) domain (I172T) (Fig. 2A) (*21*). In both strains, we used the previously characterized gene 7 construct encoding RV NSP3 and the fluorescent protein mRuby, which facilitates identification of infected cells during imaging (*23*). Performing long-term Ca^2+^ imaging of infected MA104-GCaMP6s monolayers revealed that SA11-G10-OSUv elicited a greater number of ICWs than did SA11-G10-OSUa (Fig. 2B), suggesting that NSP4 is a critical determinant of ICWs.

### The C-terminal domain of NSP4 is responsible for the differing ICW phenotypes of OSUv and OSUa

Through sequencing, we found that NSP4-OSUv and NSP4-OSUa differ at only three amino acids at positions 49, 138, and 172 (Fig. 2A and fig. S1). To determine which of these changes were responsible for the differing ICW phenotypes, we generated an additional three strains of recombinant SA11. Each of these encoded NSP4 from OSUa, but with one of the three amino acids reverted to the OSUv sequence in turn. Only the T172I reversion was sufficient to recover the ICW phenotype (Fig. 2B), suggesting that the C-terminal domain of NSP4 may play a role in activating the release of ADP to drive ICWs. We next aimed to determine whether the C-terminal domain of NSP4 acted directly upstream of ICWs or whether the change at amino acid 172 indirectly attenuated ICWs. We reasoned that the changes in the C-terminus of NSP4 may indirectly influence waves by (i) decreasing viral replication, (ii) decreasing the expression of NSP4, (iii) altering the expression of other viral proteins (*25*), or (iv) changing the trafficking dynamics of NSP4 (*32*).

To determine whether the strains lacking ICWs had reduced replication kinetics, which may indirectly cause a decrease in ICWs, we generated growth curves for each of the recombinant SA11 strains in MA104 cells. We observed no reduction in viral replication among the non–wave-inducing strains SA11-G10-OSUa, A49V, or S138P when compared to the wave-inducing strains (Fig. 2C). While there was no difference in the viral yield between 1 and 6 hpi, SA11-G10-OSUa yield was higher at 9, 12, and 24 hpi than that of SA11-G10-OSUv. Thus, the reduction in waves associated with G10-OSUa was not likely a result of an overall decrease in the rate of viral replication. We next examined the relative levels of NSP4 between viral strains. There was no difference in the level of NSP4 detected by Western blot between SA11-G10-OSUv and SA11-G10-OSUa, although levels trended higher for SA11-G10-OSUa (Fig. 2D and fig. S2A). Hence, the reduction in waves could not be attributed to a reduction in the level of NSP4.

Last, we aimed to assess whether NSP4-OSUv and NSP4-OSUa differed in cellular localization or trafficking. NSP4 is first synthesized in the ER, where it is cotranslationally inserted into the ER membrane. NSP4 conducts ER Ca^2+^ into the cytoplasm, which activates autophagy (*20*, *21*, *33*). NSP4, in association with SEC24 homolog A, COPII coat complex component (Sec24) and microtubule-associated protein light chain 3 (LC3), then exits the ER in coat protein complex II (COPII) vesicles that eventually associate with viroplasms (*33*, *34*). We aimed to determine whether ER exit may occur at a different time after infection between NSP4-OSUv and NSP4-OSUa. For these studies, we developed a line of MA104 cells (MA104-ER-GFP) that constitutively express green fluorescent protein (GFP) anchored to the transmembrane domain of Sec61β, functioning as a fluorescent marker of the ER. We infected MA104-ER-GFP cells with OSUv, OSUa, SA11-G10-OSUv, or SA11-G10-OSUa at an MOI of 0.1 and fixed at 12 hpi, as this is around the time that SA11-G10-OSUv begins to robustly activate ICWs. We did not detect a difference in the proportion of NSP4-OSUv and NSP4-OSUa that colocalized with GFP, suggesting that no major difference in the amount of NSP4 retained in the ER membrane at 12 hpi (Fig. 2, E and F). Similarly, we found no difference in the percentage of NSP4 colocalized with immunofluorescently tagged Sec61β nor with NSP2 as a marker of viroplasms, the target of NSP4 after it leaves the ER (Fig. S3). Therefore, the diminished ICW phenotype associated with the OSUa NSP4 could not be attributed to slowed replication kinetics, decreased NSP4 levels, nor major differences in NSP4 trafficking.

### Ca^2+^ signaling within infected cells is associated with the ICW phenotype

Previous studies have implicated intracellular Ca^2+^ dysregulation in the initiation of ICWs (*35*). We sought to determine whether dysregulation of Ca^2+^ signaling within the infected cell may differ between cells infected with a wave-inducing versus a non–wave-inducing strain. The cytosolic Ca^2+^ traces from cells infected with the wave-inducing SA11-G10-OSUv and SA11-G10-T172I seemed to show more marked dysregulation relative to strains associated with fewer ICWs (Fig. 2G). The strains associated with fewer ICWs also show fewer intracellular Ca^2+^ spikes (Fig. 2H). However, ADP signaling is expected to have both a paracrine and autocrine effect. Thus, a virus that induces fewer ICWs would be expected to induce fewer Ca^2+^ spikes in the infected cell due to reduced autocrine signaling. To determine whether the strains that induce fewer ICWs may be independently inducing fewer intracellular Ca^2+^ spikes, we used the LLC-MK2 line of monkey kidney epithelial cells. LLC-MK2 cells do not express the P2Y1 receptor and, therefore, do not show ICWs during RV infection (*24*). The difference in intracellular Ca^2+^ spikes within infected cells persisted in this cell line, suggesting a P2Y1-independent difference in intracellular Ca^2+^ signaling (Fig. 2H). Furthermore, across the RV strains, the average number of Ca^2+^ spikes within infected LLC-MK2 cells exhibited a positive correlation with the average number of ICWs in MA104 cells (*R*^2^ = 0.97, *P* = 0.002; Fig. 2I). Together, these results show that P2Y1-independent Ca^2+^ signaling within the infected cell predicts the ICW phenotype and, therefore, may be involved in ICW initiation.

### NSP4 is sufficient to induce ICWs

**Fig. 3.**
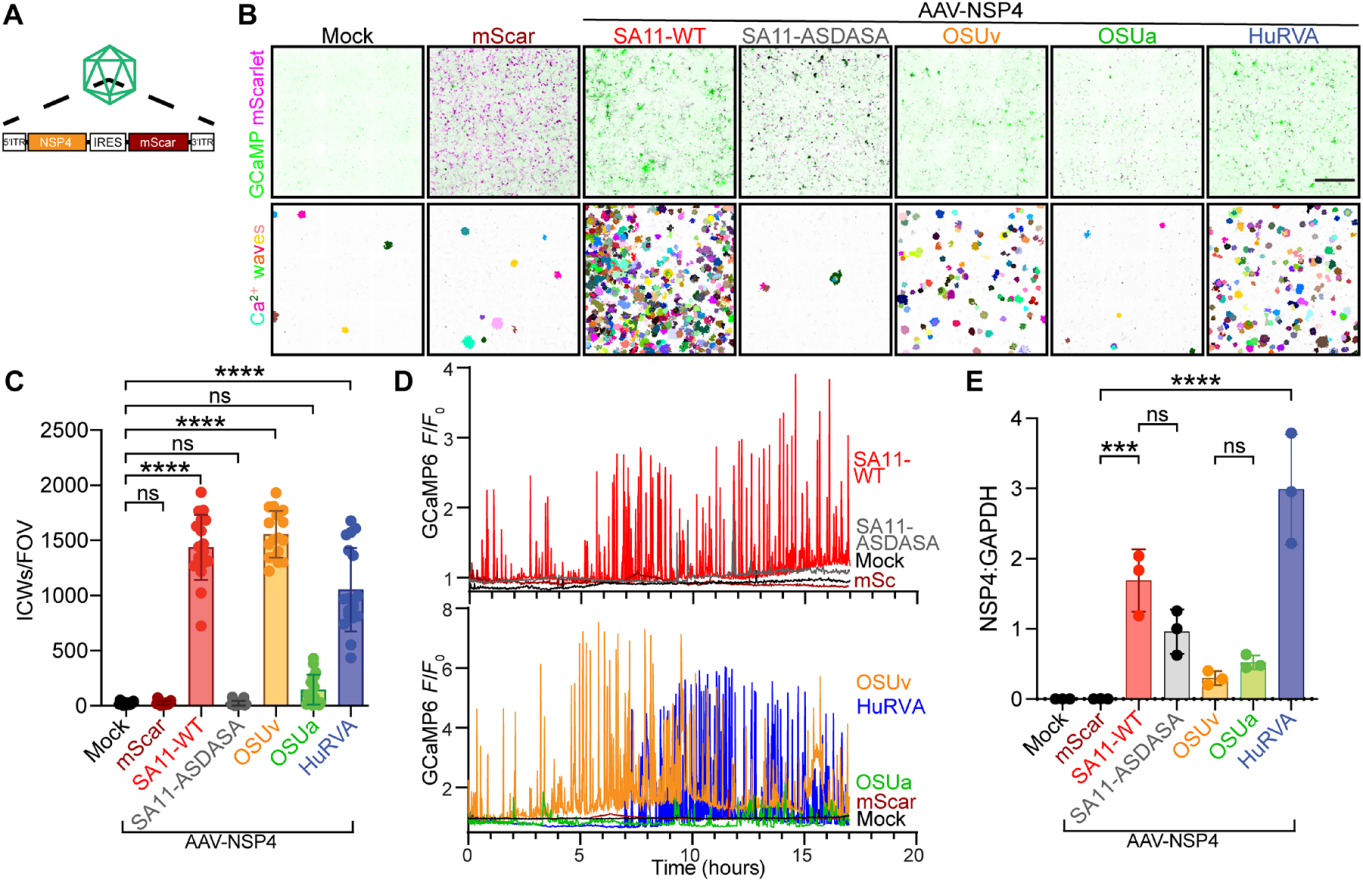
NSP4 is sufficient for ICWs. (**A**) Schematic representation of AAV vectors used to recombinantly express mScarlet (mScar) as a reporter alone or with SA11 NSP4-WT, the SA11 NSP4-ASDASA viroporin mutant, or NSP4 from strains OSUv, OSUa, or a group A human RV (HuRVA). ITR, inverted terminal repeat; IRES, internal ribosomal entry site. (**B**) mScarlet signal (magenta) overlaid on maximum intensity projections of GCaMP6s signal (green) during 30 min of imaging beginning 60 hours after transduction (top row). Segmented ICWs were arbitrarily pseudocolored to visualize and distinguish individual signaling events (bottom row). (**C**) Comparison of the number of ICWs detected during 18 hours of imaging following transduction with the indicated AAV constructs. Monolayers were imaged every 1 min for 30 min, beginning 52 hours after transduction. Data were pooled from three biological replicates. ICW counts for technical replicates were normalized to the average number of ICWs detected across all conditions within a given biological replicate. Normalized ICW counts were compared by Kruskal-Wallis, followed by Dunn’s multiple comparison tests. (**D**) Representative intracellular Ca^2+^ traces from cells mock treated or transduced with the indicated AAV construct for mScarlet or NSP4s. (**E**) NSP4 detected by Western blot in MA104 cells transduced with the indicated AAVs. NSP4:GAPDH ratios were compared by one-way ANOVA (*P* < 0.001), followed by Student’s *t* test with Šidák correction. ***P < 0.001 and ****P < 0.0001.

Given the observed segregation of the ICW phenotype with NSP4, we aimed to determine whether NSP4 would be sufficient to induce ICWs outside of the context of an RV infection. To recapitulate the high levels of NSP4 expression characteristic of RV infection, we turned to adeno-associated virus (AAV) vectors. We generated constructs encoding NSP4s from different RV strains along with a fluorescent reporter (AAV2-SA11-NSP4-mScarlet; Fig. 3A) and transduced MA104-GCaMP6s cells before performing long-term Ca^2+^ imaging. Detection of mScarlet fluorescence confirmed transduction (Fig. 3B, top row, magenta). Segmentation and projection of ICWs detected in 30 min for each stitched field of view illustrated an upregulation of ICWs associated with SA11-NSP4 (Fig. 3B, bottom row). ICW quantitation revealed that SA11-NSP4 transduction elicited a greater number of ICWs relative to AAV2-mScarlet alone or nontransduced controls (Fig. 3C). This is consistent with the intracellular Ca^2+^ traces from representative cells that were mock treated or transduced to express mScarlet alone or wild type SA11 NSP4 (SA11-WT) (Fig. 3D, top). We also generated constructs encoding NSP4 from both OSUv and OSUa. Similar to our findings from our recombinant RV strains, OSUv NSP4 induced a greater number of ICWs than OSUa NSP4 (Fig. 3C). Likewise, intracellular Ca^2+^ traces from representative cells suggest that AAV-expressed OSUa NSP4 induces fewer intracellular Ca^2+^ signals than OSUv NSP4 (Fig. 3D, bottom). Western blot and image densitometry confirmed the expression of NSP4 from all of the AAV constructs (Fig. 3E and fig. S2B). Together, these results show that ICWs are an intrinsic feature of NSP4.

### Intact viroporin activity is necessary for NSP4-induced ICWs

Expression of NSP4 by AAV2 transduction offered a flexible platform to explore the mechanistic link between NSP4 and ICWs. NSP4 is a known viroporin that conducts Ca^2+^ out of the ER in infected cells (*20*). Given the noted correlation between the frequency of Ca^2+^ signals within infected cells and the frequency of ICWs, we hypothesized that the reduced ICW count associated with NSP4-OSUa may be due to a reduced capacity to disrupt cytosolic Ca^2+^ within the infected cell. To further test this hypothesis, we generated an AAV construct encoding a mutant NSP4 that lacks viroporin activity. This mutant, dubbed the “NSP4-ASDASA” mutant, harbors missense mutations that alter six key residues within the predicted amphipathic domain (IFNTLL ➔ ASDASA) (*21*). We attempted to rescue RV strains with the ASDASA NSP4 mutant via reverse genetics but were unsuccessful. This is consistent with previous observations suggesting that NSP4-mediated Ca^2+^ dysregulation is critical for RV replication (*33*). The AAV platform circumvents this limitation, allowing high expression of NSP4 without viral replication. Transduction of MA104-GCaMP6s cells with an AAV encoding NSP4-ASDASA and a fluorescent reporter showed no difference in ICWs relative to those transduced with the fluorescent reporter alone (Fig. 3, B and C). This is further illustrated in the intracellular Ca^2+^ traces from representative cells transduced to express SA11 NSP4-WT or the NSP4-ASDASA mutant (Fig. 3D, top). This defect in ICW induction by the NSP4-ASDASA mutant was not due to diminished protein expression or posttranslational processing as Western blot analyses showed that NSP4-ASDASA was expressed to similar levels as NSP4-WT (Fig. 3E) and endoglycosidase H (EndoH) treatment confirmed that the NSP4-ASDASA mutant is both expressed and glycosylated in the ER similar to NSP4-WT (fig. S2C). This finding supports the hypothesis that viroporin activity is necessary for NSP4-induced ICWs.

### NSP4 from species A human RV induces ICWs

The NSP4 viroporin shows strong conservation across species A RVs. Thus, given that NSP4s from both porcine and simian RV strains were sufficient to induce ICWs in MA104 cells and human RV was sufficient to induce ICWs in HIO monolayers, we hypothesized that the NSP4 from a species A human RV strain would likewise be sufficient to induce ICWs. To test this hypothesis, we transduced MA104-GCaMP6s monolayers with an AAV encoding NSP4 from a human RV strain. As predicted, the NSP4 variant from human RV was sufficient to induce ICWs (Fig. 3C), showing that triggering ICWs is a conserved feature of NSP4, including that from human RV.

### NSP4 viroporin activity drives transcriptional changes

Previous studies have shown that RV infection induces broad transcriptional changes within the intestinal epithelium (*36*). How RV instigates these changes remains an outstanding question. To determine whether NSP4 and its resultant Ca^2+^ signaling affect transcriptional regulation, we performed bulk RNA sequencing on monolayers transduced with the AAVs described above. To differentiate the effects of ICWs and intracellular Ca^2+^ signals, we included cells transduced with SA11 NSP4 and treated with the P2Y1 inhibitor, BPTU, which blocks ICWs (*23*). Transcriptional changes in BPTU-treated monolayers should therefore represent the effects of NSP4-induced intracellular Ca^2+^ signals rather than those dependent on ICWs.

**Fig. 4.**
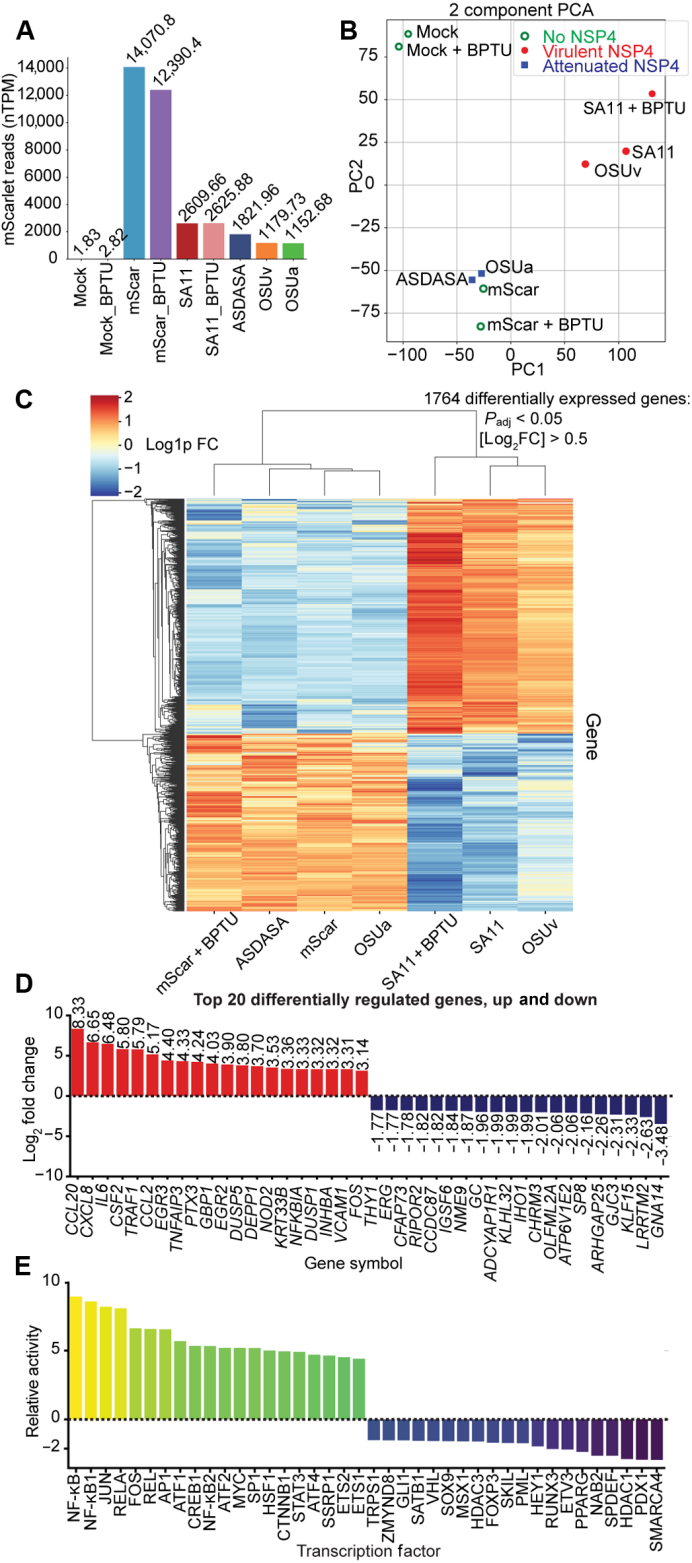
NSP4 viroporin activity drives transcriptional changes consistent with innate immune activation. MA104 cells were transduced with 50,000 genome copies of AAV encoding the indicated NSP4 and the fluorescent protein mScarlet. RNA was extracted at 36 hours after transduction. (**A**) Normalized transcripts per million (nTPM) read counts of mScarlet, as a surrogate of AAV transgene expression. (**B**) Two-component principal components analysis plot. (**C**) Heatmap of differentially regulated genes between ICW-inducing and non–ICW-inducing AAV-NSP4 variants and controls. (**D**) Top 20 differentially regulated genes. (**E**) Top 20 differentially active transcription factors in monolayers transduced with ICW-inducing AAV-NSP4 variants versus those transduced with non-ICW inducing variants and vehicle controls.

Quantification of the mScarlet reporter confirmed transduction of all constructs, albeit with varying degrees of efficiency (Fig. 4A). Two-component principal components analysis of transcript counts revealed three primary clusters: mock-transduced controls, samples transduced with the vehicle alone or with attenuated NSP4, and samples transduced with virulent NSP4 (Fig. 4B). OSUa NSP4 clustered with NSP4 ASDASA and the mScarlet (vehicle) control, whereas OSUv NSP4 clustered with SA11 NSP4. The BPTU-treated SA11 NSP4 sample showed more similarity to the untreated SA11 or OSUv NSP4 samples than the ASDASA or OSUa NSP4 samples, suggesting that the ICWs are not the major contributor to the observed transcriptional changes. Instead, this suggests that the intracellular Ca^2+^ dysregulation from NSP4 is the critical effector of the transcriptional changes induced by ICWs.

Further analysis revealed a total of 1764 differentially expressed genes between samples transduced with no/attenuated NSP4 versus those transduced with virulent NSP4 (*P*_adj_ < 0.05, [log_2_FC] > 0.5, where FC is the fold change; Fig. 4C), with many of the most highly up-regulated genes relating to innate immunity, including *CCL20*, *CXCL8*, *IL6*, *CSF2*, and *TRAF1* (Fig. 4D). This up-regulation was not seen in samples transduced with NSP4 OSUa or the NSP4 ASDASA mutant. Using the univariant linear model method to calculate transcription factor enrichment scores, we found that nuclear factor κB (NF-κB) was the most strongly up-regulated transcription factor in response to NSP4 (Fig. 4E and fig. S4A). Further, bulk RNA sequencing from MA104 cells infected with either the simian SA11 or human Ito strains of RV showed a similar activation of NF-κB (fig. S5, A and B). Gene set enrichment analysis revealed additional gene ontology terms overrepresented in the samples transduced with virulent viroporins, including genes involved in protein deubiquitination, ER overload, and protein kinase R-like endoplasmic reticulum kinase (PERK) regulation (fig. S4B). Together, these results show that NSP4 induces the expression of NF-κB–regulated genes through a viroporin activity–dependent mechanism.

### NSP4 drives differences in Ca^2+^ signaling and swelling in RV-infected HIOs

**Fig. 5.**
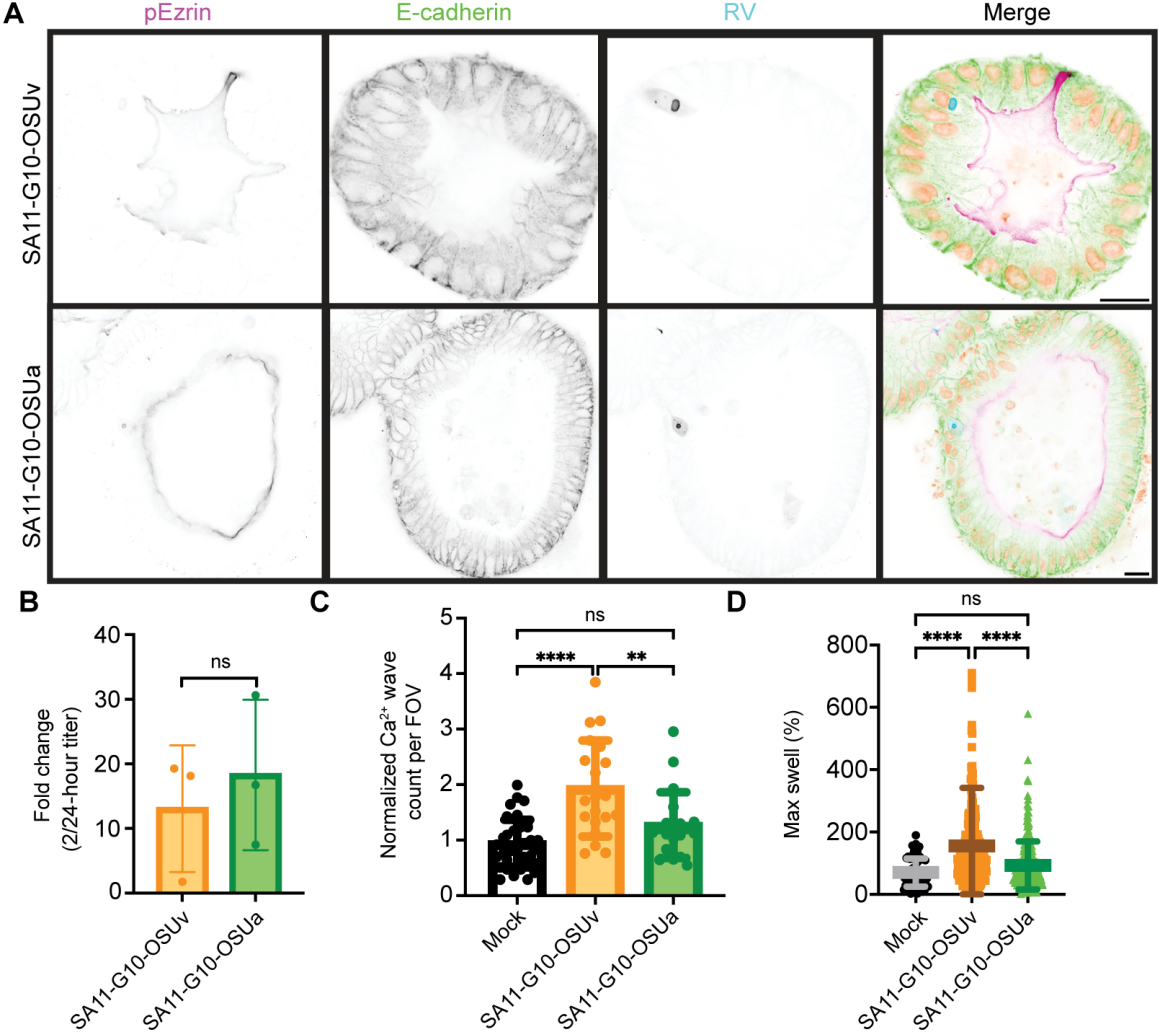
HIOs infected with SA11-G10-OSUv and SA11-G10-OSUa support replication and show distinct pathogenic markers. (**A**) Representative immunofluorescence images of three-dimensional HIOs infected with SA11-G10-OSUv and SA11-G10-OSUa at 18 hpi. Scale bars, 20 μm. (**B**) Results from yield assay showing comparable yield of SA11-G10-OSUv and SA11-G10-OSUa in three-dimensional HIOs. Represented as fold change in plaque-forming units per milliliter between 2 and 24 hpi. (**C**) ICWs detected in HIO-GCaMP6s monolayers infected with the indicated viruses. Monolayers were imaged every 1 min from 8 to 24 hpi. Data were pooled from three biological replicates. (**D**) Percent increase between initial and maximum cross-sectional area in three-dimensional HIOs during infection with mock inoculum, SA11-G10-OSUv, or SA11-G10-OSUa. Data were combined from three biological replicates. For (C) and (D), groups were compared using Kruskal-Wallis, followed by Dunn’s multiple comparison tests. **P < 0.01 and ****P < 0.0001.

While MA104 cells provided a valuable tool for initial investigation, they are of neither human nor intestinal origin. HIOs provide an RV-susceptible model system that more closely recapitulates the human gastrointestinal epithelium. We first verified that HIOs were susceptible to the recombinant strains of SA11 expressing OSUv or OSUa NSP4 by performing yield assays and immunofluorescence. After a 1-hour inoculation in suspension and 24 hours of growth thereafter, RV antigen was detected by immunofluorescence in HIOs inoculated with either strain of recombinant SA11 (Fig. 5A). Further, RV yield at 24 hpi confirmed replication of both strains in HIOs (Fig. 5B).

Next, we next aimed to characterize the ICW phenotype associated with each strain in HIOs. For these experiments, we used monolayers generated from the jHIO-GCaMP6s line (*37*). We infected differentiated monolayers with RV and performed live Ca^2+^ imaging for 18 hpi. ICW analysis revealed that, similar to our findings in MA104 cells, SA11-G10-OSUv infection induced more ICWs than SA11-G10-OSUa in HIO monolayers (Fig. 5C).

HIOs also provided a valuable model system to investigate fluid secretion, a key aspect of RV pathogenesis. We performed swelling assays to compare the secretory activity associated with each virus (*29*). SA11-G10-OSUv was associated with a larger percent increase in cross-sectional area than SA11-G10-OSUa (Fig. 5D), suggesting that the wave-inducing NSP4 from OSUv is associated with more fluid secretion than the non–wave–inducing NSP4 from OSUa.

### NSP4 influences RV pathogenesis in mice

**Fig. 6.**
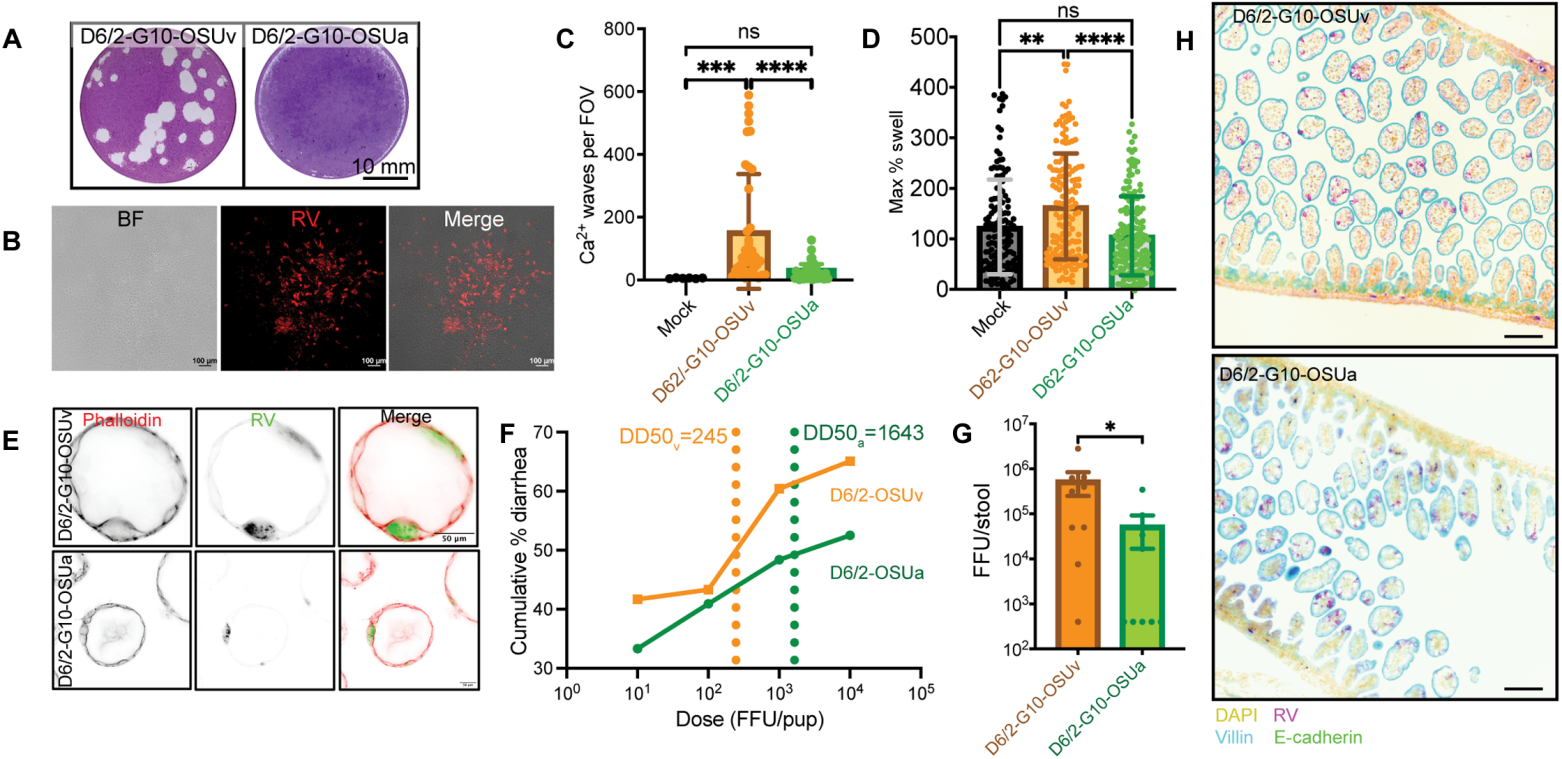
NSP4 is associated with ICWs, organoid swelling, disease, and shedding in a murine-like RV background. (**A**) Representative images of plaque assays showing plaque clearance at 3 days postinfection in MA104 monolayers infected with D6/2-G10-OSUv, but nonlytic plaques in monolayers infected with D6/2-G10-OSUa. (**B**) Representative bright-field (BF) and immunofluorescence image of monolayer infected with D6/2-G10-OSUa (red) showing the presence of infected foci despite the lack of plaque clearance. (**C**) Quantitation of ICWs in MA104-GCaMP6s monolayers imaged every 1 min from 8 to 24 hpi. Data were combined from three biological replicates. (**D**) Maximum percent swell in three-dimensional mouse intestinal organoids (Balb/c jejunum) infected with the indicated RV strains. For (C) and (D), groups were compared using Kruskal-Wallis, followed by Dunn’s multiple comparison tests. (**E**) Detection of RV antigen (green) and filamentous actin (red) in three-dimensional mouse intestinal organoids. (**F**) Litters of CD-1 mice were infected with the indicated dose of D6/2-G10-OSUv (*n* = 40 pups across four litters) or D6/2-G10-OSUa (*n* = 63 pups across four litters) and followed for 5 days postinfection. The percentage of pups to develop diarrhea during the 5-day period was used to plot the cumulative incidence curves and estimate the dose required to produce disease in 50% of animals [diarrhea dose 50 (DD50)] for each virus. (**G**) Infectious particles detected in stool from animals infected with 10^4^ FFU at 3 days postinfection by fluorescent-focus assay. *n* = 9 pups per condition. Mean titers compared by Mann-Whitney test. (**H**) Representative immunofluorescence images of RV (magenta), 4′,6-diamidino-2-phenylindole (DAPI) (yellow), villin (cyan), and E-cadherin (green) detected in intestinal epithelium from infected pups at 2 days postinfection. Scale bars, 100 μm. *P < 0.05, **P < 0.01, ***P < 0.001, and ****P < 0.0001.

Our results in HIOs suggested that NSP4-OSUv and NSP4-OSUa differ in their ability to induce fluid secretion. Because fluid secretion is an integral aspect of RV pathogenesis, we sought to determine whether the disease phenotype associated with NSP4-OSUv and NSP4-OSUa would differ in vivo. We generated novel viruses using the recently developed D6/2 murine-like RV reverse genetics system (*15*). We packaged viruses with either NSP4-OSUa (D6/2-G10-OSUa) or NSP4-OSUv (D6/2-G10-OSUv). Both strains grew to similar titers in MA104 cells; however, while the D6/2-G10-OSUv variant formed clear plaques in MA104 cells, D6/2-G10-OSUa failed to do so (Fig. 6A). Nevertheless, detection of RV antigen by immunofluorescence showed that plaque-like foci still formed in monolayers infected with D6/2-G10-OSUa (Fig. 6B). Thus, NSP4-OSUa in a D6/2 background impaired MA104 cell cytotoxicity in a manner we did not observe in the SA11 background. Similar to SA11, however, live Ca^2+^ imaging of infected MA104-GCaMP6s monolayers showed that OSUv-NSP4 was associated with a higher frequency of ICWs than OSUa-NSP4 in the D6/2 background (Fig. 6C). To assess the secretory phenotype associated with the D6/2 strains, we first performed swelling assays in jejunal murine intestinal organoids (jMIOs). jMIOs infected with D6/2-G10-OSUv showed a larger percent increase in cross-sectional area than those infected with D6/2-G10-OSUa (Fig. 6D), recapitulating our findings from SA11-infected HIOs. Immunofluorescence provided further evidence of successful infection (Fig. 6E).

Next, we determined the diarrhea dose 50 (DD50) for both viruses in the CD-1 neonatal mouse model of RV diarrhea by infecting individual litters with 10^1^, 10^2^, 10^3^, or 10^4^ focus-forming units (FFU) per pup. The estimated DD50 for D6/2-G10-OSUa was 1643 FFU, while it was only 245 FFU for D6/2-G10-OSUv (Fig. 6F), representing a 6.7-fold decrease in the DD50 for the OSUv NSP4 variant. We also compared the quantity of infectious viral particles shed from litters infected with 10^5^ FFU per pup by performing focus-forming assays on stool samples collected at 3 days postinfection. Animals infected with D6/2-G10-OSUv shed, on average, 10-fold more virus than animals infected with D6/2-G10-OSUa (Fig. 6G). Immunofluorescence staining of intestinal sections extracted from a subset of these animals provided further evidence of infection (Fig. 6H). Overall, we found that relative to D6/2-G10-OSUa, D6/2-G10-OSUv was associated with more swelling in HIOs, more viral shedding in vivo, and a left-shifted diarrhea dose curve consistent with a higher propensity to cause disease. These differences stemmed from only three polymorphisms in NSP4.

## DISCUSSION

ICWs driven by the release of purines from virus-infected cells constitute a major component of RV pathogenesis and disease (*23*). While blocking ICWs through pharmacological inhibition of the purinergic receptor, P2Y1, reduces disease severity in RV-infected mice, whether ICWs are an RV-intrinsic determinant of virulence is not known. Further, how RV induces the release of purines to drive ICWs remains unclear. To address these gaps in knowledge, we used the RV reverse genetics system, AAV vectors, live imaging, and in vivo models to investigate the mechanism through which RV induces ICWs. Using recombinant RVs expressing NSP4 from either a virulent or attenuated porcine strain, we showed that the ICW phenotype segregated with NSP4 and predicted virulence. Using AAV vectors to express NSP4 in isolation, we established the sufficiency of NSP4 for ICWs. Further, our studies indicate that NSP4’s function as a viroporin, conducting Ca^2+^ from the ER into the cytosol of infected cells, is required for the induction of ICWs. Given our recent finding that ICWs enhance RV spread, this introduces a mechanism through which the manipulation of Ca^2+^ within the infected cell promotes viral replication and pathogenesis (*24*). Given the existence of Ca^2+^-conducting viroporins and pore-forming toxins among other viral families and pathogens, these findings have implications beyond RV.

A local increase in cytosolic Ca^2+^ is a known instigator of ICWs (*38*, *39*). However, how the elevation of cytosolic Ca^2+^ is sensed and translated into the release of purinergic signals remains an open question. Recent work established that intracellular Ca^2+^ levels modulate the activation of pannexin-1, a well-characterized adenosine 5′-triphosphate (ATP)–permeable plasma membrane channel (*40*). Elevated intracellular Ca^2+^ activates calcium- and calmodulin-dependent protein kinase II, which phosphorylates pannexin-1 and increases its ATP conductance. Alternatively, nucleotide release may occur not in direct response to elevated Ca^2+^ but as a result of disrupted Ca^2+^ regulated homeostatic processes. Ca^2+^ regulation of the actin cytoskeleton is one such process, conserved across biological kingdoms (*41*). NSP4 Ca^2+^ mobilization causes actin remodeling that may result in altered mechanical properties of the plasma membrane, which could potentiate the nucleotide release mechanism, as mechanical stimulation of the plasma membrane remains one of the most extensively studied ICW instigators (*42*–*44*).

Through RNA sequencing, we found that ICW-inducing NSP4 variants also activate NF-κB, activating protein 1 (AP1), Myc, and a variety of other host transcription factors that are not activated by NSP4 variants with diminished Ca^2+^ conductance and ICW induction. The activation of NF-κB and other innate immune markers suggests that viroporin-mediated Ca^2+^ signaling represents a homeostasis altering molecular process or stress-associated molecular pattern, both of which produce similar signals and host responses (*45*–*49*). Similar to NSP4, the severe acute respiratory syndrome coronavirus (SARS-CoV) E protein conducts Ca^2+^, activates an innate immune response, and contributes to virulence (*50*). While we have yet to determine whether the SARS-CoV E protein is sufficient to induce ICWs, SARS-CoV-2 infection activates pannexin-1 channels, which can conduct purines and instigate ICWs (*51*–*53*). Beyond viruses, bacterial pore-forming toxins can elevate cytosolic Ca^2+^ levels (*54*). While this usually manifests as a rapid, monophasic influx of extracellular Ca^2+^, pore-forming toxins with the capacity to open and close can elevate Ca^2+^ with multiphasic kinetics (*54*). Further, streptolysin O, hemolysin A, lysteriolysin, and leukotoxins can induce the release of Ca^2+^ from intracellular stores, similar to viroporins (*54*). Alpha hemolysin from pathogenic *Escherichia coli* induces interleukin-6 (IL-6) and IL-8 expression in a Ca^2+^-dependent manner (*55*). Here, we found that IL-6 and IL-8 were two of the three most strongly up-regulated transcripts induced by viroporin-competent NSP4s. Thus, while the manipulation of cytosolic Ca^2+^ levels may benefit pathogens in a pathogen-specific manner, they also likely elicit a conserved host innate immune response triggered by similarities in the dynamics of their Ca^2+^ dysregulation.

While NSP4-mediated intracellular Ca^2+^ dysregulation caused both transcriptional changes and ICWs, we have yet to determine the relationship between these two effects. Treatment with BPTU, a known inhibitor of RV ICWs (*23*), did not ameliorate the transcriptional changes following NSP4 expression. This suggests that these changes are not dependent on ICWs but, instead, arise in direct response to NSP4-mediated intracellular Ca^2+^ dysregulation. However, to produce a level of NSP4 that mimicked an RV infection, we had to transduce cells with AAV-NSP4 at a high multiplicity. Despite the high multiplicity, the expression of the fluorescent reporter and immunoblot analysis indicate that only a subset of cells expresses an amount of NSP4 comparable to an RV infection. Nevertheless, nearly every cell is expressing some NSP4, thus likely experiencing some degree of viroporin-mediated Ca^2+^ disruption. In this setting, the transcriptional effects specific to ICWs may be masked by the NSP4-generated increase in Ca^2+^ signals. In a low-multiplicity RV infection, there would be far more cells receptive to ICWs than expressing NSP4. We would predict the ICWs to have a proportionally greater effect in this setting. Furthermore, RV NSP1 antagonizes antiviral responses, which may offset some of the NSP4-induced transcriptional changes within infected cells (*56*, *57*). This antagonism is not predicted to occur in uninfected neighboring cells, further augmenting the relative effect of ICWs. NSP1’s inhibition of the host immune response is less effective during heterologous infections (*58*, *59*). Less effective immune suppression by a heterologous NSP1 and diminished calcium signaling may have synergized to result in D6/2-G10-OSUa’s inability to clear plaques in MA104 cells. While this warrants further investigation, this synergism would not be expected to occur during a homologous infection in mice, where NSP1-mediated immune suppression would be more effective. Hence, the attenuation of D6/2-G10-OSUa in vivo is likely independent of the plaque phenotype in MA104 cells.

Unlike NSP1, NSP4 is well conserved among group A RVs from different primary host species, particularly within the viroporin domain (*60*, *61*). While NSP4s from group B and group C RVs have weaker sequence homology, they are predicted to maintain functional homology and viroporin activity (*62*). In our studies of the OSUv and OSUa NSP4s, we found that reversion of the 172nd amino acid from the threonine in the OSUa NSP4 to the isoleucine in the OSUv NSP4 was sufficient to recover an OSUv-like ICW phenotype. How this residue influences Ca^2+^ mobilization remains an outstanding question, and an isoleucine at position 172 has not been characterized in NSP4s from any other RV strains. Previous work has shown that this region of NSP4 binds both host proteins, including tubulin, and the viral protein VP6 (*63*–*65*). Since the differential Ca^2+^ phenotypes of NSP4-OSUv and NSP4-OSUa persist when NSP4 is expressed alone, they cannot be dependent on interactions between NSP4 and other RV proteins. Thus, amino acid 172 must either influence Ca^2+^ signaling via an interaction with a host protein or by directly modulating Ca^2+^ conductance through the viroporin domain. Our initial hypothesis was that this residue was involved in a protein-protein interaction that influenced the rate of NSP4 exit from the ER into COPII vesicles and, eventually, the autophagosome-like compartment that colocalizes with RV viroplasms (*33*, *34*). In this way, the NSP4 cytoplasmic tail could indirectly reduce the NSP4 intracellular signals by regulating the amount of NSP4 in the ER with access to the ER Ca^2+^ store. However, we detected no difference in the amount of NSP4 colocalized with general ER markers in cells infected with OSUv versus OSUa. Nevertheless, we cannot rule out that the NSP4 cytoplasmic tail sequence regulates a differential distribution among ER subdomains, such as ER exit sites, where its channel function may be reduced. While the structure of full-length NSP4 is not yet solved, it would offer insight into the role of the C-terminal domain in NSP4-mediated Ca^2+^ mobilization.

In addition to distinct ICW phenotypes, the NSP4 from OSUv was associated with a left-shifted diarrhea dose curve and more viral shedding in vivo, suggesting higher pathogenicity relative to the NSP4 from OSUa. It is possible that the propensity to cause diarrhea stems from accelerated growth kinetics. We previously showed that the inositol 1,4,5-trisphosphate receptor activation associated with ICWs facilitates viral spread in vitro (*24*) and this may also contribute to the increased shedding associated with the ICW-prone OSUv-NSP4 observed here. However, OSUv-NSP4 was also associated with increased swelling in our HIO model, despite no major detectable difference in viral yield. Because these experiments last only 12 to 18 hours and there is no trypsin in the culture medium to activate viral progeny, viral spread would not be a contributing factor in HIO swelling. This suggests that ICWs may have pathogenic consequences both related to and independent of their ability to promote viral spread.

By infecting GCaMP6s-expressing HIOs with the virulent human RV strain, Ito, or the LAV strain, RV1, we found a similar association between ICW frequency and virulence for human RV. The virulent strain induced a higher frequency of ICWs despite reduced infectivity. While we have yet to attribute these differences to NSP4, these results suggest that ICWs are an important facet of human RV pathogenesis. The NSP4 sequence from the virulent Ito and attenuated RV1 strains are similar, sharing 97.1% identity at the amino acid level, but do not have the same differences as found between the NSP4s from OSUv and OSUa virus strains. Thus, the difference between OSUv and OSUa NSP4s is certainly not the only changes that can result in the production of fewer ICWs, which underscores the importance of future studies to phenotype the ICW potential of NSP4s from different strains, both during bona fide infections and recombinant expression of NSP4. Nevertheless, similar to the porcine and simian NSP4s, NSP4 from a human RV strain was sufficient to induce ICWs. Thus, the ability to trigger ICWs is a conserved intrinsic feature of NSP4 from multiple group A RVs. Similar to most viral proteins, however, the effects of NSP4 may be indirectly affected by other viral processes. Variations in other viral proteins that slow replication kinetics, diminish immune antagonism, or, otherwise, truncate the infective cycle would be expected to attenuate ICWs by reducing the level of NSP4 or lifespan of the infected cell. Hence, a reduction in ICWs may predict attenuation in vivo that is not directly attributable to NSP4. In the work detailed here, we opted to focus on OSUv and OSUa NSP4s as the high sequence homology made for more efficient phenotype mapping. On the basis of these results, we predict that our ongoing studies to unravel the relationship between Ca^2+^ signaling and viral pathogenesis will find a similar role for NSP4-induced Ca^2+^ signaling in human RV virulence.

NSP4 viroporin activity may also promote pathogenesis by producing ER stress. NSP4 viroporin activity releases ER calcium, which, if excessive enough, could substantially reduce ER Ca^2+^ levels to drive the accumulation of unfolded proteins (*66*–*68*). Many pathogens are associated with the activation of the unfolded protein response, which contributes to pathogenesis and disease (*69*, *70*). Whether this benefits the pathogen or represents an incidental effect of infection remains unclear and likely varies between pathogens. Gene ontology analysis of the RNA sequencing data included here detected an enrichment of transcripts related to the PERK-mediated unfolded protein response and ER overload in cells expressing viroporin-competent NSP4 variants. However, such a strong enrichment was not observed in cells infected with RV at low multiplicity, wherein there was a more prominent increase in DNA replication/cell division pathways. This difference is likely due to the relatively few infected cells in this experimental setup and contrasts with the AAV transduction studies wherein most/all of the cells are expressing NSP4. Together, these results suggest that the ER overload observed in our experiments expressing NSP4 alone may have been the result of a combined insult from NSP4-mediated ER Ca^2+^ depletion and prolonged overexpression of both NSP4 and the mScarlet reporter.

While the studies presented here revealed mechanistic insight into the role of NSP4 in ICW induction, they had a few key limitations. First, mimicking NSP4-mediated Ca^2+^ signaling without NSP4 expression would help to more firmly establish viroporin-mediated Ca^2+^ signaling as the trigger of ICWs. However, we have been unable to establish a system to do this. The Ca^2+^ traces from RV-infected or NSP4-expressing cells suggest that the signals induced by NSP4 are discrete and dynamic, with amplitudes that trend higher than signals detected in mock-infected cells. While optogenetic tools allow for temporal control of Ca^2+^ signals, most rely on the activation of endogenously expressed voltage-gated channels to produce meaningful signals (*71*). RV, however, predominantly infects enterocytes, which are nonexcitable. Hence, we have been able to mimic the temporal dynamics of NSP4 using optogenetic constructs, but the signal amplitude is much lower than that of NSP4-induced signals. In future work, we aim to more comprehensively characterize NSP4-induced intracellular calcium signals and develop techniques to more effectively mimic them. Another limitation stemmed from our transcriptional studies, which were performed in MA104 cells, an excellent cell model for RV replication and the study of NSP4-generated Ca^2+^ signaling and ICWs. Ultimately, the use of more relevant model systems, such as HIOs or in vivo, with sufficient NSP4 expression will be needed to dissect the role these Ca^2+^ signaling pathways play in RV pathogenesis and host responses. Nevertheless, our findings from the transcriptomics studies relate to elementary processes that are likely conserved across most mammalian cell types and establish an experimental framework to compare the host responses between different groups of viroporins.

Overall, this work identifies viroporin-mediated disruption of cytosolic Ca^2+^ as a novel signifier of pathogenic invasion and a key contributor to RV virulence. Not only viroporin activity induced transcriptional changes associated with canonical innate immunity, but also ICWs have been previously shown to promote viral spread (*24*). ICW induction was associated with increased viral shedding and disease in vivo, suggesting that ICWs may confer a selective advantage to RV that, when combined with innate immune antagonism by other viral proteins (*17*, *56*), supersedes the deleterious effects predicted by innate immune activation. This implicates NSP4 and the pathways that respond to the associated Ca^2+^ aberrations as attractive therapeutic targets. Manipulation of NSP4 in LAV strains may allow for attenuation without substantial changes to structural proteins that constitute key RV antigens, promoting immunity without fulminant disease. Drugs that target the NSP4 viroporin itself, Ca^2+^-responsive proteins, or P2Y1-mediated ICWs may be useful antiviral and antidiarrheal therapeutics. Given that other enteric viruses, including caliciviruses and picornaviruses, encode Ca^2+^-conducting viroporins, these findings may have implications for human pathogens beyond RV (*72*, *73*).

## MATERIALS AND METHODS

### Cell culture

MA104 (American Type Culture Collection, CRL-2378.1) and LLC-MK2 (American Type Culture Collection, CCL.7) cells were cultured in high-glucose Dulbecco’s modified Eagle’s medium (DMEM) with 10% fetal bovine serum and 1× antibiotic/antimycotic (Invitrogen) at 37°C with 5% CO_2_. Cells were passaged every 2 to 3 days.

### Rotaviruses

The parental OSUv and OSUa strains (gifts from L. Svensson), Ito strain (gift from M. Estes), and RV1 strain (gift from S. Ramani) were propagated by inoculating MA104 cells at a low MOI (~0.01) for 1 hour before rinsing and switching to DMEM with Worthington’s trypsin (1 μg/ml) for 3 days or until >90% of the monolayer showed cytopathic effect. Stocks then underwent three freeze-thaw cycles, and aliquots were activated with Worthington’s trypsin (10 μg/ml) at 37°C for 30 min before use. Virus stock titers were determined by plaque assay or fluorescence focus-forming assay on MA104 cells. All stocks were stored at −80°C between uses. The recombinant SA11 and D6/2 strains were generated using established protocols for reverse genetics (*15*, *74*).

### Human intestinal organoids

All HIOs used in these studies were acquired from the Gastrointestinal Experimental Model Systems Core at the Texas Medical Center Digestive Diseases Center. HIOs were maintained in Wnt3a, R-spondin-3, Noggin, epidermal growth factor (WRNE) culture medium as previously described (*23*, *29*). HIOs were differentiated for 3 to 5 days before RV infections as previously described (*29*, *75*). For Ca^2+^ imaging, three-dimensional HIOs were digested into a single-cell suspension by incubating in TrypLE (Gibco) for 5 min and vigorously pipetting before dispersing onto collagen IV–coated imaging bottom plates (Greiner) (*76*). After 24 hours, monolayers were switched from WRNE to differentiation medium. Monolayers were seeded 72 hours before RV infection or imaging. For RV infection, RV stocks were diluted in differentiation medium, and a single score was introduced along the diameter of the monolayer using a 23-gauge needle. Monolayers were inoculated for 2 hours, rinsed, and then incubated in differentiation medium.

### Live calcium imaging

MA104, LLC-MK2, and HIO lines stably expressing the cytosolic calcium indicator GCaMP6s were generated using lentivirus transduction as previously described (*37*, *54*, *55*). Monolayers were seeded in eight-well, imaging-bottom chamber slides (Ibidi) and allowed to grow for 48 hours or until confluent. Monolayers were mock or RV infected at a low MOI (0.005 to 0.05) for 1 hour. For HIO studies, mock inoculum included mock-infected, trypsinized MA104 lysate equalized to the volume of virus stock included in RV infections. A single scratch was introduced down the length of HIO monolayers immediately after addition of the inoculum to allow infection (*76*). The inoculum was removed, monolayers were rinsed, and medium was switched to phenol-free imaging medium (FluoroBrite, Gibco). Eight fields of view were selected at random for each well. Using a 20× Plan Apo objective, 50-ms exposures at 50% light source power, images were acquired on the fluorescein isothiocyanate (FITC) line every 1 min, and tetramethyl rhodamine isothiocyanate line every 10 min from 7 to 25 hpi. Slides were maintained in a stage-top, temperature- and humidity-controlled chamber with 5% CO_2_. For experiments involving RV strains that did not express a fluorescent reporter, following live imaging, monolayers were fixed with 4% formaldehyde, and RV-infected cells were identified via immunofluorescence as described below.

### Image analysis

For the analysis of image series from long-term calcium imaging experiments, we used the open-source platform FIJI (*77*). FITC (GCaMP6s) and tetramethyl rhodamine isothiocyanate (RV mRuby) acquisitions were split into separate stacks and processed individually. Each image in the FITC time series was subtracted from the next to determine the change in fluorescence at each pixel between acquisitions (Δ*F*). A minimum Δ*F* threshold was applied (+600 relative fluorescent units) before detecting features based on a minimum size (10^4^ contiguous micrometers), which represent ICWs. The total number of ICWs within each field of view was compared across treatment conditions.

### Generation and validation of custom anti-NSP4 antibodies

Antibodies against NSP4 amino acids (aa)114-135 were produced commercially (ABclonal Technology, Woburn, MA) (Table 1). Briefly, a synthetic peptide of SA11 NSP4 aa114-135 (DKLTTREIEQVELLKRIYDKLT-C) was synthesized and validated by high-performance liquid chromatography and mass spectrometry. Preimmune serum was collected before three guinea pigs were immunized five times over 84 days with the synthetic peptide. Immunized antisera were collected and purified by antigen affinity chromatography on day 96. For validation, four confluent monolayers were infected with SA11-G7-mRuby, SA11-G10-OSUv, SA11-G10-OSUa, or mock inoculum at an MOI of 5 before lysis at 9 hpi. Technical replicates were pooled and separated on duplicate SDS–polyacrylamide gel electrophoresis gels before transferring to nitrocellulose membranes. One membrane was stained with postimmune sera, and one was stained with an equal volume of preimmune sera. Membranes were then stained with equivalent concentrations of an anti–guinea pig fluorescently conjugated secondary antibody before imaging on a fluorescent scanner (fig. S6).

**Table 1.** Antibodies, stains, and dyes.

Type	Target	Species	Supplier	Catalog no./ID	Dilution
Primary	SA11 RV NSP4 aa114-135	Guinea pig	AB Clonal	Custom	1:1000
Primary	SA11 RV NSP4	Mouse	Gift from Estes and colleagues (*91*)	Monoclonal antibody B4/2, purified	1:500
Primary	SA11 RV	Rabbit	Gift from Estes and colleagues (*33*)	Rabbit pool 42	1:5000
Primary	SA11 RV vNSP2	Mouse	Gift from Estes and colleagues(*92*)	vNSP4 monoclonal antibody	1:2000
Primary	Phospho-Ezrin	Rabbit	Cell Signaling Technologies	3726	1:500
Primary	E-cadherin	Rabbit	GeneTex	1004433	1:200
Primary	Sec61β	Rabbit	Invitrogen	PA3-015	1:500
Primary	GAPDH	Mouse	Novus	NB-600-502	1:5000
Primary, conjugated	Villin	Mouse	Santa Cruz Biotechnology	Sc-58897	1:500
Secondary	Antirabbit Alexa Fluor 488	Donkey	Rockland	611-741-127	1:1000
Secondary	Antirabbit Alexa Fluor 555	Donkey	Rockland	606-142-129	1:1000
Phalloidin 555	Actin	*Amanita phalloides*	Thermo Fisher Scientific	A34055	1:1000
DAPI	Double-stranded DNA		Invitrogen	R37606	2 drops/ml

### Immunofluorescence

Culture medium was removed from MA104 or HIO monolayers before rinsing with 1× phosphate-buffered saline (PBS). Monolayers were fixed for 30 min with 4% formaldehyde and then washed three times with 1× PBS. HIO monolayers were incubated in 50 mM NH_4_Cl for 30 min following fixation. Monolayers were blocked for 1 hour at room temperature or overnight at 4°C with 5% bovine serine albumin in 1× PBS and then permeabilized for 30 min with 0.01% Triton X-100 in 1× PBS. Monolayers were then incubated in primary antibodies (Table 1) diluted to the indicated concentrations in 1× PBS overnight at 4°C, washed four times with 1× PBS, and incubated with the indicated fluorescently conjugated secondary antibodies for 1 hour at room temperature. Monolayers were again washed four times with PBS and maintained at 4°C in 1× PBS until imaged.

### Western blot

Confluent mock-infected, RV-infected, or AAV-transduced MA104 cell monolayers seeded in 24-well culture plates were incubated in 150 μl of radioimmunoprecipitation assay buffer [50 mM tris, 150 mM NaCl, 1% NP-40, 0.5% sodium deoxycholate, 0.1% sodium dodecylsulfate, and 1 EDTA-free protease inhibitor tablet (Sigma-Aldrich) (pH 7.4)] and subjected to one freeze-thaw cycle. Where indicated, a 30-μl aliquot of lysate was transferred to a clean 1.5-ml microcentrifuge tube and incubated with 1 μl of Endo-H (New England Biolabs) for 45 min in a 37°C bead bath. The volume (20%) of 5X SDS–polyacrylamide gel electrophoresis sample buffer was added to each lysate before incubating at 100°C for 10 min. Thirty microliters of lysate was loaded per well of a precast 4 to 20% polyacrylamide gel. Gels were loaded into an electrophoresis rig and run at 120 V for 75 min. Protein was transferred to a nitrocellulose membrane using a semidry transfer apparatus. Membranes were allowed to dry completely, rehydrated for 2 min with 1× PBS, and blocked for 1 hour using Intercept Blocking Buffer (LI-COR). Primary antibodies (Table 1) were diluted in 1× PBS with 0.2% Tween 20 and 10% Intercept Blocking Buffer by volume and incubated overnight. Membranes were washed four times with 1× PBS and 0.2% Tween 20 before incubating secondary antibodies diluted 1:20,000 in 1× PBS for 1 hour. Membranes were rinsed an additional four times with 1× PBS and 0.2% Tween 20 and once with 1× PBS, then dried, and imaged on a LI-COR Odyssey CLx scanner.

### RV growth curves

Confluent MA104 cell monolayers were infected with the indicated strains of RV for 1 hour at an MOI of 0.5. The inoculum was removed, and monolayers were washed with 1× PBS before replacing with serum-free high-glucose DMEM. At 1, 3, 6, 9, 12, and 24 hpi, monolayers underwent three freeze-thaw cycles, were transferred to 1.5-ml microcentrifuge tubes, centrifuged to remove cell debris, and incubated for 45 min at 37°C with Worthington’s trypsin (10 μg/ml). RV yield was assessed by plaque assay as described below.

### Adeno-associated viruses

AAV vectors were synthesized and packaged commercially (VectorBuilder). NSP4 sequences and accession numbers for the AAVs were SA11 clone 3 (AAC61867), OSUa (BAA13728.1), and human RV group A (ABC25504.1). Amino acid differences between OSUa and OSUv NSP4s are shown in Fig. 2A, and the SA11 NSP4-ASDASA mutant was previously described (*21*). For imaging experiments, 5 × 10^4^ MA104-GCaMP6s cells were seeded on eight-well imaging bottom chamber slides (Ibidi). Twelve hours after seeding, monolayers were transduced with 50,000 genome copies of AAV per cell diluted in high-glucose DMEM with 10% fetal bovine serum. Transduction medium was left on monolayers for 24 hours, rinsed once with 1× PBS, and then switched to phenol-free cell culture medium (FluoroBrite+, Gibco). Live Ca^2+^ imaging was performed from 32 to 50 hours after transduction as described above. For Western blots, 24-well cell culture plates were seeded with 1.5 × 10^6^ MA104 cells per well (Corning) and transduced 12 hours after seeding. Lysates were harvested with 150 μl of radioimmunoprecipitation assay buffer and processed as described above.

### Swelling assays

HIOs were switched to differentiation medium 2 days after passage. After 3 to 5 days of differentiation, HIOs were removed from basement membrane matrix using 500 μl of ice-cold 1× PBS per well and spun for 4 min at 4°C. Supernatant was removed, and pellets were resuspended in 1 ml of 1× PBS before a second spin. Pellets were then resuspended in 200 μl of inoculum consisting of 5 × 10^6^ plaque-forming units (PFU) of trypsin-activated RV or trypsinized uninfected MA104 cell lysate diluted in CMGF^−^ for 1 hour at 37°C. HIOs were pelleted, washed once with PBS, resuspended in 25% basement membrane matrix diluted in phenol-free differentiation medium, and plated on an eight-well imaging bottom chamber slide (LabTek) precoated with a thin layer (~4 μl) of basement membrane matrix. The slide was then loaded into a humidified stage-top incubation chamber maintained at 37°C with 5% CO_2_ on a Nikon TiE inverted epifluorescence microscope. Nikon NIS-Elements software was used to acquire bright-field images of 50+ HIOs per condition every 5 min for 18 hours and 555-nm epifluorescence images every 20 min for experiments using mRuby-tagged RV strains. FIJI was used to measure the initial and maximal cross-sectional area of each HIO and to calculate the percent change (maximum percent swell). MIOs were infected in suspension for 2 hours 1 day after passage using the same method.

### Yield assay in HIOs

Differentiated HIOs were removed from basement membrane matrix using ice-cold PBS, pelleted, washed in 1× PBS, and resuspended in 200 μl of RV or mock inoculum as described above. After 2 hours, HIOs were pelleted and washed; half were harvested for the 2-hour titer, and half were resuspended in 30 μl of basement membrane matrix until the 24-hour time point. To determine the viral yield, HIOs and supernatant underwent three freeze-thaw cycles before trypsin activation and titration by plaque assay.

### Plaque assay

RV infected lysates (cells and supernatant) underwent three freeze-thaw cycles and activation with Worthington’s trypsin (10 μg/ml) for 30 min at 37°C before 10-fold serial dilution. MA104 cells were grown to confluency in six-well plates and switched to serum-free medium for 24 hours before infection. Wells were infected with 200 μl of each serial dilution in duplicate for 1 hour at 37°C with gentle shaking every 15 min to ensure even distribution. Monolayers were rinsed once with 1× PBS, and medium was replaced with a semisolid overlay consisting of 1.2% Avicel in serum-free DMEM supplemented with *O*-(diethylaminoethyl) dextran and Worthington’s trypsin (1 μg/ml). After 72 hours, overlay was removed, and monolayers were stained with crystal violet.

### Fluorescence focus assay

A 96-well cell culture plate was seeded with 1.5 × 10^4^ MA104 cells per well and grown to confluency. Twenty-four hours before infection, plates were switched to serum-free high-glucose DMEM. RV stocks were activated with Worthington’s trypsin (10 μg/ml) for 30 min at 37°C before fourfold serial dilution in serum-free high-glucose DMEM. Each well was inoculated with 100 μl for 1 hour at 37°C. The inoculum was removed, wells were rinsed with 1× PBS, and medium was replaced. At 18 hpi, the medium was removed, and wells were rinsed with 1× PBS before fixation with 100% methanol for 20 min at 4°C. Wells were rinsed three times with 1× PBS, incubated overnight with 50 μl of primary antibody solution, rinsed another three times, and then incubated for 1 hour at room temperature with secondary antibody solution. The secondary antibody solution was removed, and nuclei were stained with NucBlue Fixed Cell Stain per the manufacturer’s protocol. Monolayers were washed and maintained in 100 μl of 1× PBS per well. A 10×/0.30 Plan Fluor objective on a Nikon Eclipse Ti epifluorescence microscope was used to acquire a 7.5-mm by 7.5-mm stitch image covering the entirety of each well. Rolling ball background subtraction and thresholding were performed in FIJI before quantifying the number of infected cells per well via the particle analysis plug-in.

### RNA sequencing

A total of 1.5 × 10^5^ MA104-GCaMP6s cells were seeded per well in a 24-well cell culture plate. For experiments involving AAV, AAV encoding NSP4 and a fluorescent reporter was added 12 hours after seeding to indicated wells (5 × 10^4^ genome copies per cell) as described above. At 72 hours after transduction, the plates were imaged to verify expression of the fluorescent reporter before column-based isolation of RNA (RNeasy Mini Kit, QIAGEN) with on-column DNA digestion. For experiments using RV infection, SA11, Ito, or mock inoculum (MOI of 0.50) was added for 1 hour at 48 hours after seeding in technical triplicate. RNA quality and quantity were assessed by fluorimetry (Qubit Extended Range Assay Kit, Thermo Fisher Scientific) and stored at −80°C. Isolation of mRNA was achieved via polyadenylate enrichment before library preparation (Ultra II Directional RNA library prep kit, New England Biolabs). Libraries were sequenced on an Illumina NovaSeq with 40 M 150-bp paired-end reads per sample.

Read quality was assessed using FastQC v0.12.1 (*78*) and MultiQC v1.19 (*79*). Reads were aligned to the *Chlorocebus sabaeus* genome assembly 1.1 accessed via Ensembl (GCA_000409795.2) (*80*) using STAR aligner v2.7.11a (*81*). Expression of the AAV transgene was estimated by quantitating mScarlet transcripts using the kallisto v0.46.1 pseudoaligner (*82*). Read counts were compared using PyDESeq2 (*83*) v0.4.4 and GSEApy (*84*) v1.1.1. To generate transcription factor enrichment scores, *t* values generated using PyDESeq2 were analyzed against the CollectTRI network using the Univariate Linear Model method from decoupleR (*85*, *86*).

### Mouse intestinal organoids

All MIOs used in these studies were generated and maintained by the Gastrointestinal Experimental Modal Systems Core at the Texas Medical Center Digestive Diseases Center following established protocols (*87*–*89*). For all studies, we used mouse intestinal organoids generated from the jejunum of two separate adult BALB/c mice, which were passaged 25 to 45 times before experimentation. MIOs were maintained in Advanced DMEM/F12 (Gibco) supplemented with 1× GlutaMAX (Gibco), 0.01 M Hepes, penicillin-streptomycin (100 U/ml; Gibco), 20% R-spondin-1 (Rspo-1) conditioned medium, 5% Noggin conditioned medium, 1 mM N2 supplement (Invitrogen), B27 supplement (Invitrogen), 1 mM N-acetylcysteine (Sigma-Aldrich), and recombinant murine epidermal growth factor (50 ng/ml; Invitrogen). Every 3 to 4 days, MIOs were passaged by removing maintenance medium, adding 300 μl of 0.05% trypsin, and mechanically breaking up basement membrane matrix by gently pipetting up and down before incubating for 5 min at 37°C. Five hundred microliters of CMGF^−^ was then added, and the suspension was vigorously pipetted 20× to promote crypt fission. The suspension was pelleted, washed once with 1× PBS, and then resuspended in basement membrane matrix before replating.

### DD50 in RV mouse model

All experiments were approved by the Institutional Animal Care and Use Committee at the Baylor College of Medicine (protocol AN-6903) and performed in accordance with the National Institutes of Health Guide for the Care and Use of Laboratory Animals. For all mouse experiments, litters including female and male animals in naturally occurring ratios were used. CD-1 dams with litters of males and females in natural ratios were purchased from the Center for Comparative Medicine at Baylor College of Medicine. Pups were housed with their dam in standard cages in a biosafety level 2 animal facility with food diet (7922 NIH-07 mouse diet, Harlan Laboratories) and water provided ad libitum throughout the experiment. Individual litters were randomly assigned for inoculation with 10, 10^2^, 10^3^, or 10^4^ FFU per pup of D6/2 G10-OSUv or D6/2 G10-OSUa at postnatal day 3. Pups were infected by oral gavage with inoculum consisting of virus stock diluted with DMEM to a final volume of 50 μl per animal. Filter-sterilized green food-grade dye was included in the inoculum to visualize delivery to the stomach. Litters were monitored for 5 days postinfection with weights and stool assessed daily. At the time of assessment, pups were gently palpated on the abdomen to encourage defecation. Stool was scored using a four-point scale for consistency, color, and quantity as previously described (*19*). Stool was collected and stored at −80°C for later assessment of viral titer, as well as secondary scoring by a blinded observer. Pups with weights that remained >2 SDs below the mean weight of the litter were excluded from analysis. The amount of virus required to induce disease in 50% of pups (DD50) was estimated using methods previously described (*90*).

### Assessment of viral shedding

Stool from animals infected with 10^4^ FFU was dissociated by adding 40 μl of 1× PBS with Ca^2+^ and Mg^2+^ after thawing. Each sample was then sonicated for 30 min, diluted 1:100 with serum-free DMEM, and activated with Worthington’s trypsin (10 μg/ml) for 30 min at 37°C. After activation, samples were serially diluted 1:4 with serum-free DMEM. Fluorescence focus assays were performed as described above.

### Mouse intestine histology

CD-1 litters with males and females in natural ratios were inoculated with 10^5^ FFU per pup of D6/2 G10-OSUv or D6/2 G10-OSUa at postnatal day 3. At days 3, 4, and 5 postinfection, three animals were euthanized from each litter for histological examination. Following euthanasia, the small intestine was extracted, flushed with 1 ml of 1× PBS delivered via 23-gauge needle inserted into the proximal end of the duodenum, and then flushed with 10% formalin by the same method. The intestine was then rolled from the proximal to distal end and placed in a tissue cassette. The tissue cassettes were then immersed in 10% formalin overnight before switching to 70% ethanol. The samples were paraffin embedded and sectioned onto glass slides.

One slide from each animal underwent hematoxylin and eosin staining for general histopathological examination, and one was used for detection of RV by immunofluorescence. Slides used for immunofluorescence were deparaffinized and rehydrated in xylene, followed by gradient ethanol and distilled water. Slides were incubated in sodium citrate buffer [10 mM sodium citrate and 0.05% Tween 20 (pH 6.0)] for 15 min in a high-pressure pressure cooker, followed by 15 min of depressurization and cooling. The slides and buffer were then incubated on ice for 30 min and then washed in 1× PBS for 5 min. The tissue was blocked for 1 hour at room temperature with 10% normal goat serum in 1× PBS before a 24-hour, 4°C incubation with primary antibodies (Table 1) diluted in 1× PBS. Primary antibody solution was removed, slides were rinsed three times with 1× PBS for 3 min, and then secondary antibodies added for 1 hour at room temperature. Secondary antibody solution was removed and replaced with NucBlue Fixed Cell Stain for 10 min at room temperature per manufacturer’s protocol. After three additional 3-min washes with 1× PBS, excess liquid was allowed to evaporate before applying mounting medium (ProLong Gold) and coverslips. Stained tissue sections were imaged using a Zeiss AxioScan Z1 whole slide scanner with a 20×/0.80 objective.
