# Supplementary material for: Viroporin activity is necessary for intercellular calcium signals that contribute to viral pathogenesis
Source: Sci Adv. 2025 Jan 17;11(3):eadq8115. doi: 10.1126/sciadv.adq8115 (PMC11740935; doi:10.1126/sciadv.adq8115)
Supplement: Supplementary file 1 — Figs. S1 to S6 [file sciadv.adq8115_sm.pdf]

Science Advances
AAAS

Supplementary Materials for

Viroporin activity is necessary for intercellular calcium signals that contribute to viral pathogenesis

J. Thomas Gebert *et al.*

Corresponding author: Joseph M. Hyser, joseph.hyser@bcm.edu

Sci. Adv. **11**, eadq8115 (2025)
DOI: 10.1126/sciadv.adq8115

This PDF file includes:

Figs. S1 to S6

A

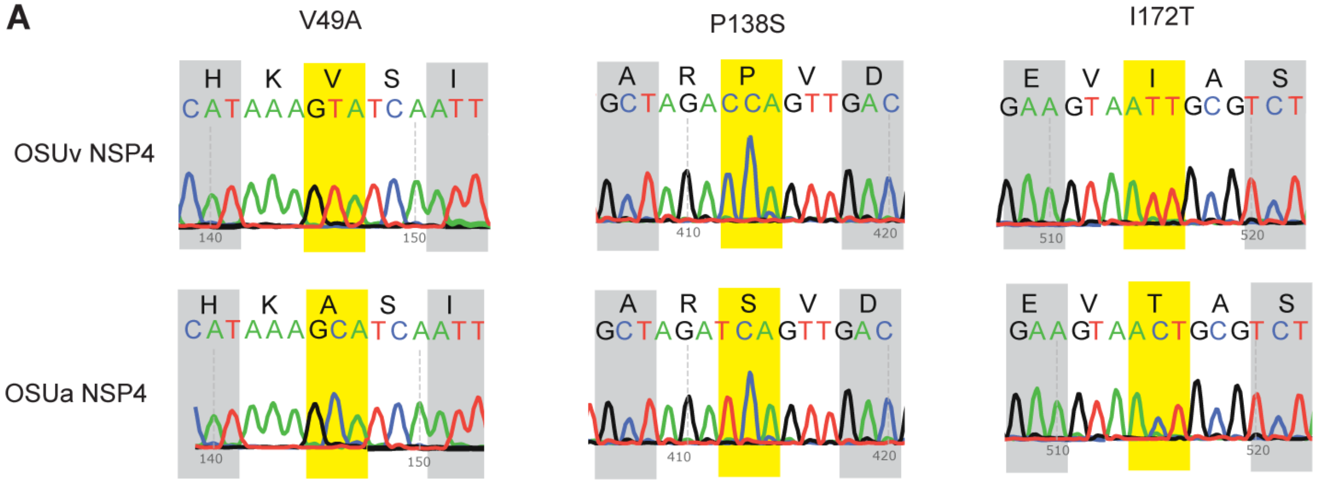

B

```
>OSUv   1  MDKLADLNYTLSVITLMNDTLHSIIQDPGMAYFPYIASVLTVLFTLHKVSIPTMKIALKTSKCSYKVIKYCMVTIINTLL
>OSUa   1  MDKLADLNYTLSVITLMNDTLHSIIQDPGMAYFPYIASVLTVLFTLHKASIPTMKIALKTSKCSYKVIKYCMVTIINTLL

>OSUv  80  KLAGYKEQVTTKDEIEQQMDRIIKEMRRQLEMIDKLTTREIEQVELLKRIHDKLAARPVDAIDMSKEFNQKNIRTLDEWE
>OSUa  80  KLAGYKEQVTTKDEIEQQMDRIIKEMRRQLEMIDKLTTREIEQVELLKRIHDKLAARSVDAIDMSKEFNQKNIRTLDEWE

>OSUv  160  SGKNPYEPSEV IASM
>OSUa  160  SGKNPYEPSEVTASM
```

Supplemental Figure S1. NSP4 from OSUv and OSUa differ by 3 amino acids. A. dsRNA from OSUv and OSUa rotavirus was isolated, reverse transcribed, amplified, and column-purified for Sanger sequencing, which revealed 3 polymorphisms in OSUa (yellow). **B**. Sequence alignment of full-length NSP4 from OSUv and OSUa showing the 3 resultant amino acid polymorphisms (red).

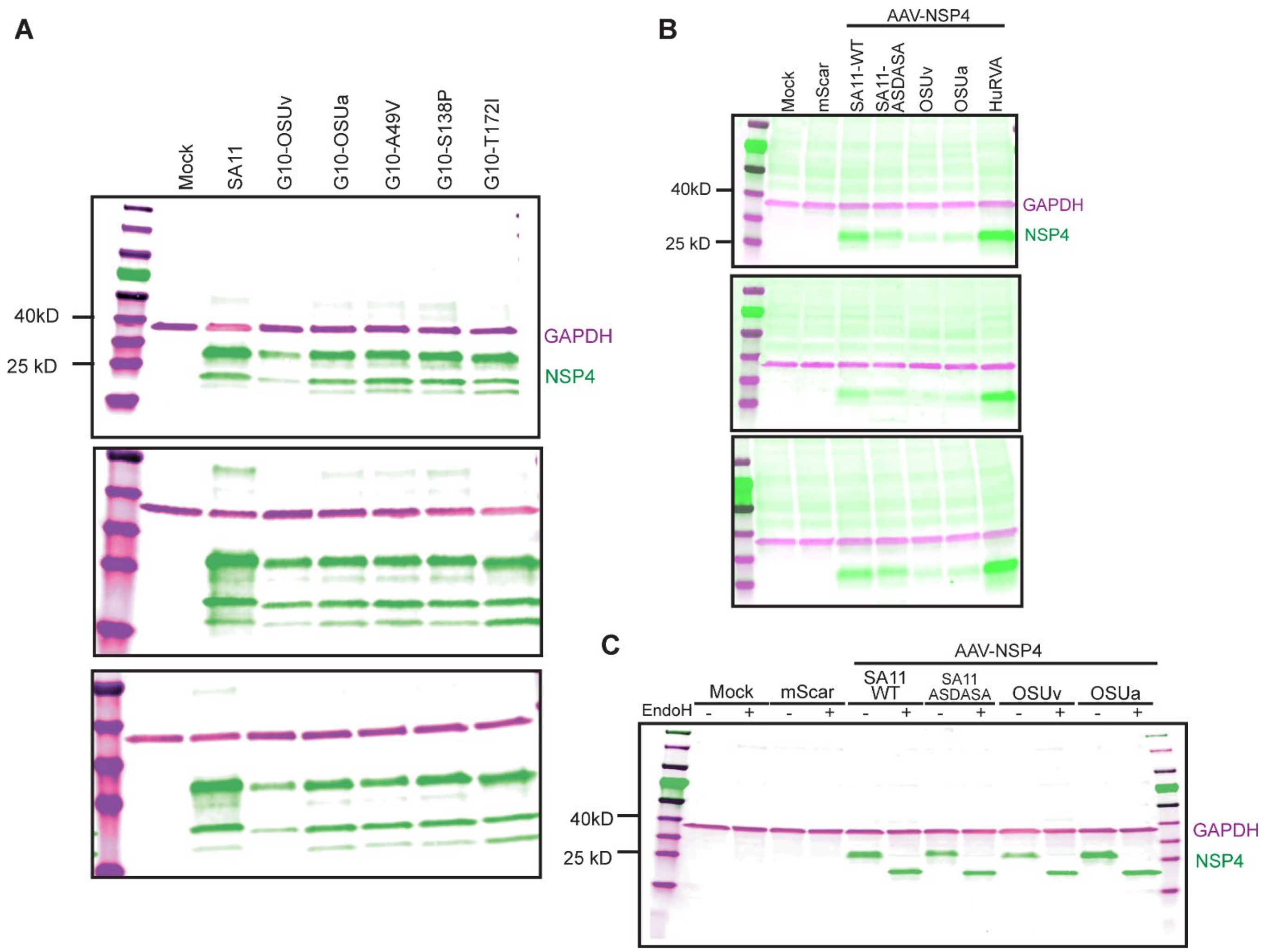

Supplemental Figure S2. Comparison of NSP4 expression from recombinant RV strains and AAV. A. Images of immunoblots for GAPDH (magenta) and NSP4 (green) detected in lysates from MA104 cells infected with the indicated strains of RV at MOI 1 at 12 hpi. Three biological replicates were run independently (vertical panels). Quantitative comparison of the NSP4:GAPDH ratio for each strain across the 3 replicates is included in main Figure 2D**. B.** Images of immunoblots for GAPDH (magenta) and NSP4 (green) detected in lysates from MA104 cells transduced with AAVs encoding the indicated constructs. Quantitative comparison of the NSP4:GAPDH ratio for each strain across the 3 replicates is included in main Figure 3E. **C.** Immunoblot for GAPDH (magenta) and NSP4 (green) detected in lysates from MA104 cells transduced with AAVs encoding the indicated constructs. To show proper NSP4 glycosylation, lysates were either untreated (-) or treated with EndoH (+) to remove N-linked glycans.

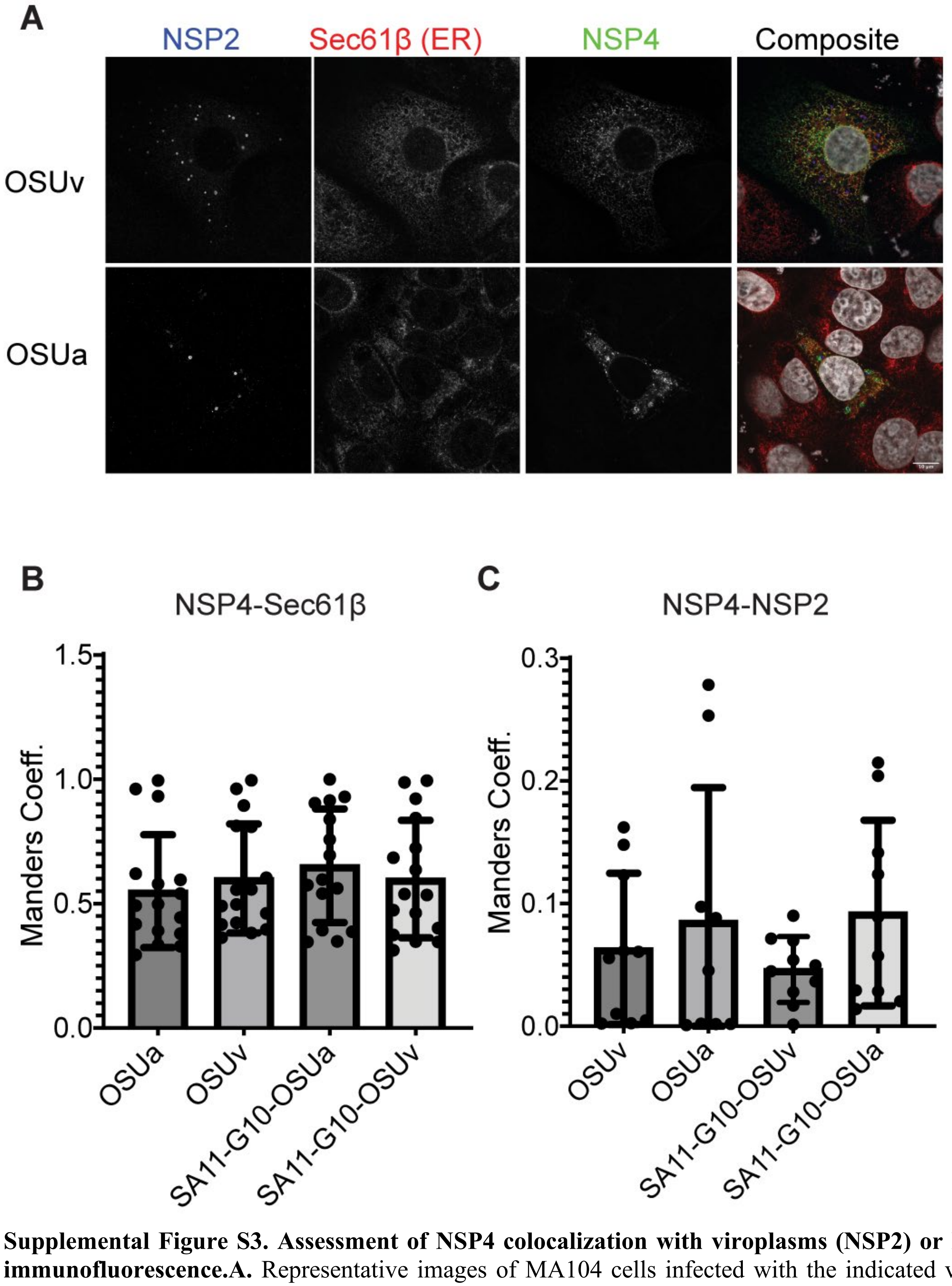

Supplemental Figure S3. Assessment of NSP4 colocalization with viroplasms (NSP2) or ER (Sec61β) by immunofluorescence.A. Representative images of MA104 cells infected with the indicated viruses at MOI 1 before fixing at 12 hpi and detecting the indicated proteins by immunofluorescence. 63X magnification. **B.** Manders coefficient estimating colocalization between OSUa and OSUv NSP4 with Sec61β by immunofluorescence. n=16 cells per condition, 3 biological replicates **C.** Manders coefficient estimating colocalization between OSUv and OSUa NSP4 with NSP2, a marker of viroplasms. n=9 cells per group, 3 biological replicates. Shapiro-Wilk test for normality ($p<0.05$ for 3/4) followed by Kruskal-Wallis ($p>0.05$).

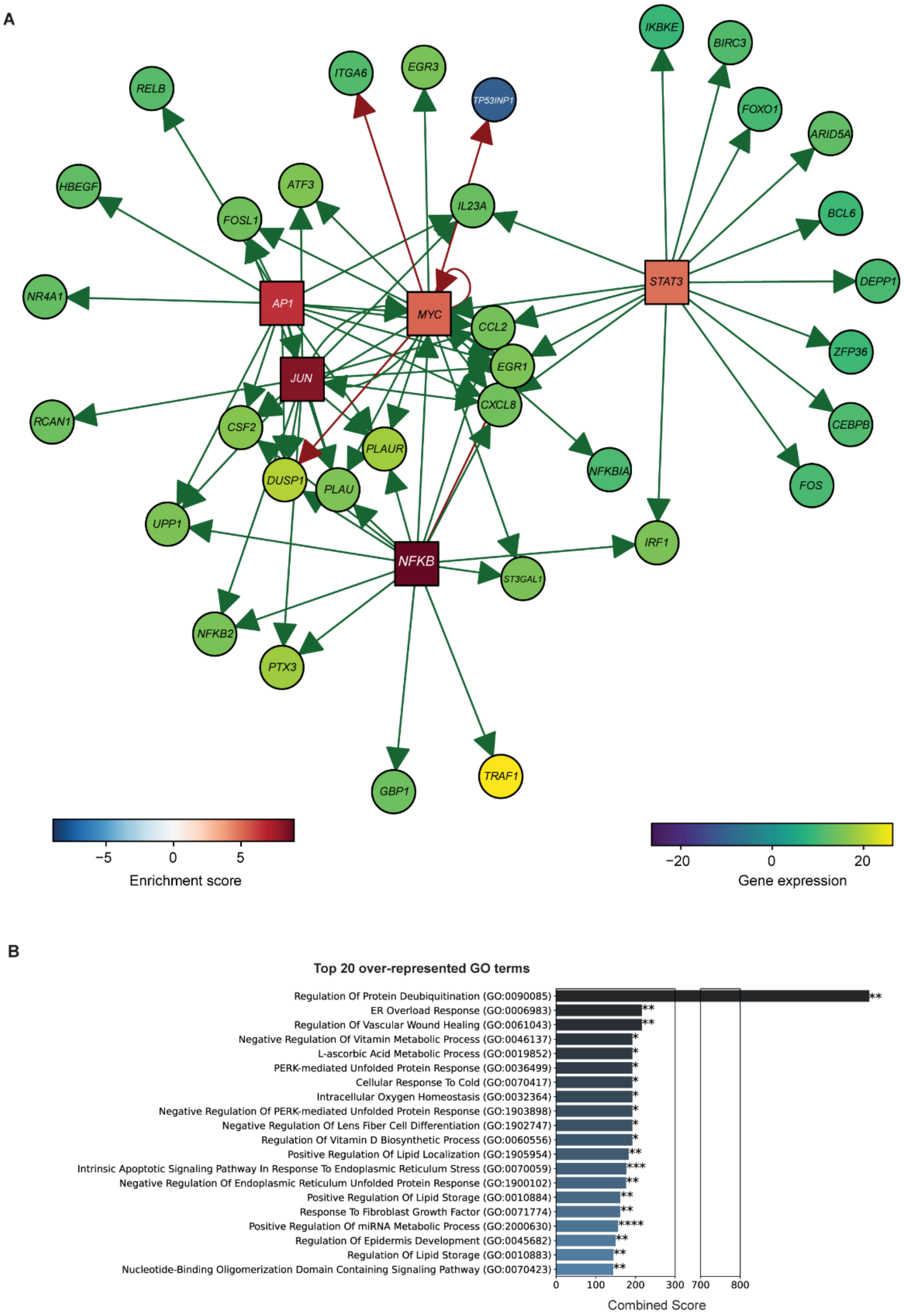

Supplemental Figure S4. Transcription factor regulatory networks and GO terms associated with the expression of viroporin-competent NSP4 variants from AAV constructs. A. Transcription factor regulatory network activity inferred from RNA sequencing in cells expressing viroporin-competent, ICW-inducing NSP4 variants (SA11 NSP4 WT, SA11 NSP4 WT + BPTU, OSUv NSP4) versus non-ICW signaling samples (mScarlet, mScarlet + BPTU, SA11 NSP4-ASDASA mutant, OSUa NSP4). Specific transcripts are represented as circular nodes with the relative expression of each indicated using an indigo-to-yellow color map (bottom right scale bar). Individual transcription factors are represented as square hubs with the relative activity of each estimated by the blue-to-red color map (bottom left scale bar). Arrows indicate the transcripts used to estimate the activity of each transcription factor (hub), with green arrows representing a positive correlation, red arrows a negative correlation. **B**. Combined scores for most significantly overrepresented gene ontology terms associated with viroporin-competent, ICW-inducing NSP4 variants.

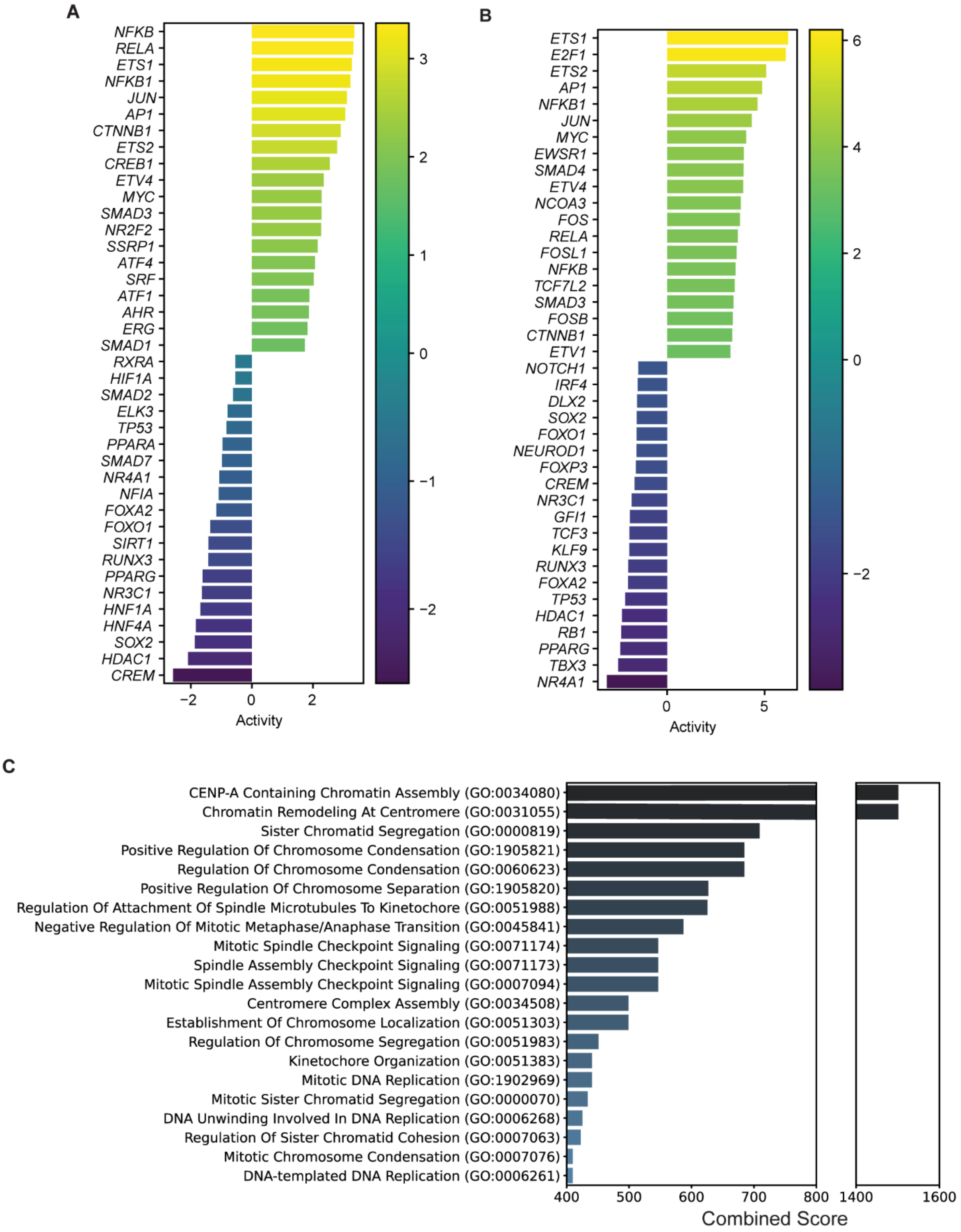

Supplemental Figure S5. Transcription factor activity and GO term analysis from RV-infected MA104 cells. A-B. Top up- and down-regulated transcription factors inferred from RNA sequencing of MA104 monolayers infected with the simian RV strain, SA11 (n=3) **(A)**. or the human RV strain, Ito (n=3) **(B)** relative to mock-infected controls (n=3). **C.** Top overrepresented GO ontology terms associated with Ito infection.

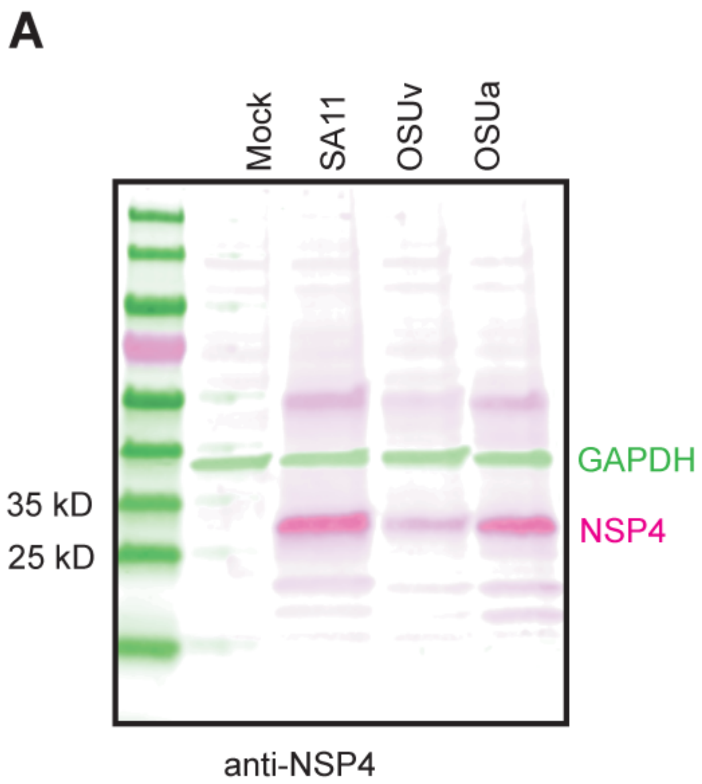

Supplemental Figure S6. Validation of custom anti-NSP4 polyclonal antibodies. Immunoblots from MA104 cell lysates after infection with mock, SA11, SA11-G10-OSUv, or SA11-G10-OSUv inoculum at MOI 5, collected at 9 hpi. 4 technical replicates were pooled and ran on two SDS-PAGE gels. One membrane was stained with the custom polyclonal antibody raised against a synthetic NSP4 aa114-135 peptide (**A**), the other with pre-immune serum collected from the immunized animal prior to NSP4 challenge (**B**). The expected 28 kD band, representing fully glycosylated NSP4, along with the sub-28 kD bands representing monoglycosylated or unglycosylated NSP4, are visualized in the gel stained with the anti-NSP4 serum.
